# Tumor-derived GCSF Alters Tumor and Systemic Immune System Cell Subset Composition and Signaling

**DOI:** 10.1158/2767-9764.CRC-22-0278

**Published:** 2023-03-09

**Authors:** Israel Matos, Maunish Barvalia, Manreet K. Chehal, A. Gordon Robertson, Iva Kulic, Jessica A.F.D. Silva, Abhinandan Ranganathan, Amy Short, Yu-Hsuan Huang, Erin Long, John J. Priatel, Salim Dhanji, Brad H. Nelson, Danielle L. Krebs, Kenneth W. Harder

**Affiliations:** 1 Department of Microbiology and Immunology, University of British Columbia, Life Sciences Institute, Vancouver, British Columbia, Canada.; 2 Canada's Michael Smith Genome Sciences Centre, BC Cancer Agency. Vancouver, British Columbia, Canada.; 3 ME Therapeutics Inc. Vancouver, British Columbia, Canada.; 4 Department of Pathology and Laboratory Medicine, University of British Columbia, Vancouver, British Columbia, Canada.; 5 Deeley Research Centre, BC Cancer, Victoria, British Columbia, Canada.

## Abstract

While immunotherapies such as immune checkpoint blockade and adoptive T-cell therapy improve survival for a subset of human malignancies, many patients fail to respond. Phagocytes including dendritic cells (DC), monocytes, and macrophages (MF) orchestrate innate and adaptive immune responses against tumors. However, tumor-derived factors may limit immunotherapy effectiveness by altering phagocyte signal transduction, development, and activity. Using Cytometry by Time-of-Flight, we found that tumor-derived GCSF altered myeloid cell distribution both locally and systemically. We distinguished a large number of GCSF-induced immune cell subset and signal transduction pathway perturbations in tumor-bearing mice, including a prominent increase in immature neutrophil/myeloid-derived suppressor cell (Neut/MDSC) subsets and tumor-resident PD-L1^+^ Neut/MDSCs. GCSF expression was also linked to distinct tumor-associated MF populations, decreased conventional DCs, and splenomegaly characterized by increased splenic progenitors with diminished DC differentiation potential. GCSF-dependent dysregulation of DC development was recapitulated in bone marrow cultures *in vitro*, using medium derived from GCSF-expressing tumor cell cultures. Importantly, tumor-derived GCSF impaired T-cell adoptive cell therapy effectiveness and was associated with increased tumor volume and diminished survival of mice with mammary cancer. Treatment with neutralizing anti-GCSF antibodies reduced colonic and circulatory Neut/MDSCs, normalized colonic immune cell composition and diminished tumor burden in a spontaneous model of mouse colon cancer. Analysis of human colorectal cancer patient gene expression data revealed a significant correlation between survival and low GCSF and Neut/MDSC gene expression. Our data suggest that normalizing GCSF bioactivity may improve immunotherapy in cancers associated with GCSF overexpression.

**Significance:**

Tumor-derived GCSF leads to systemic immune population changes. GCSF blockade restores immune populations, improves immunotherapy, and reduces tumor size, paralleling human colorectal cancer data. GCSF inhibition may synergize with current immunotherapies to treat GCSF-secreting tumors.

## Introduction

Crucial advances in cancer immunotherapy include immune checkpoint blockade (ICB) utilizing antibodies that bind inhibitory receptors such as CTLA-4 and PD-1/PD-L1, and adoptive T-cell therapies to direct T-cell responses against tumors. While responses to these therapies are successful in a subset of patients, most are transient or unresponsive (1–3). In many ways, the immune response to cancer parallels the response to chronic infection, with triggering of immune inhibitory programs leading to a state of exhaustion or suppression together with chronic inflammation (4). Given that the effectiveness of immunotherapies hinges on tumor stroma components and interactions, including changes in innate immune cell composition and activity (5, 6), it is imperative that we better understand the phagocyte regulatory pathways impacted by tumor growth if we are going to improve cancer immunotherapies.

Phagocytes in particular play a pivotal role in initiating and sustaining both innate and adaptive immune responses against tumors. Phagocytes are heterogeneous and include monocytes (Mo), macrophages (MF), dendritic cells (DC), neutrophils (Neut), which derive from common myeloid progenitors and granulocyte/MF progenitors in the bone marrow (BM). However, in chronic inflammatory conditions such as cancer, tumor, and/or stroma-derived factors including MCSF, IL6, VEGF, GCSF, and others, reprogram myelopoiesis (7) leading to development of tumor-promoting phagocytes such as Neut/myeloid-derived suppressor cells (MDSC), tumor-associated MFs (TAM), and immature DCs (8, 9).

Neut/MDSCs inhibit T cells (10, 11) and natural killer (NK) cells, recruit regulatory T cells (Treg); (12) and suppress immune responses (10–12), and elevated Neut/MDSCs correlate with poor response to ICB and adoptive T-cell therapies (13–17). On the other hand, functional tumor antigen–specific cross-presenting conventional dendritic cell (cDC) frequency correlates with better outcome and response to ICB (18, 19) while DC deficiency or immature DC accumulation leads to poor T-cell responses (20–22).

Many human cancers, including colorectal, breast, and lung, exhibit high GCSF and/or GCSF receptor (GCFR) expression (23–27). To investigate the effect of tumor-derived GCSF on immune cells, we previously used a murine mammary tumor model in which GCSF is an abundant tumor-secreted factor. This model revealed that tumor-derived GCSF is associated with perturbations in immune system development, including altered BM hemopoiesis, splenomegaly and dramatically increased multipotent splenic progenitors (28). Here, we used mouse mammary and colorectal cancer models (29), together with Cytometry by Time-of-Flight (CyTOF), to further characterize the role played by GCSF in tumor-induced immune cell compositional and signal transduction changes associated with tumor growth. This system-level characterization showed that increased GCSF dramatically altered phagocyte distribution in tumor, BM, spleen, and blood, leading to increased Neut/MDSCs, decreased cDC1s, and extramedullary hemopoiesis characterized by splenomegaly with elevated splenic progenitors with diminished DC differentiation potential. GCSF-dependent dysregulation of DC development was recapitulated in BM cultures *in vitro*. We also identified signal transduction pathways within these immune subsets associated with GCSF upregulation, including the GCSF-STAT3 axis.

Changes in the immune compartment stimulated by tumor-derived GCSF translated to increased tumor size and load, and decreased efficacy of adoptive T-cell transfer therapy. We show that GCSF and Neut/MDSC gene expression is elevated in human colorectal cancer and is associated with colorectal cancer patient outcome. Our work suggests that normalizing GCSF expression levels may be useful alone, or in combination with new immunotherapies, for tumors expressing GCSF.

## Materials and Methods

### Mice

Experiments were performed using 8–10 weeks old MMTV-neu/OT-I/OT-II (MMTV), C57BL/6, GCSFR^−/−^, BoyJ, Rag1^−/−^, and OT-I age- and sex-matched mice. Animals were housed under specific-pathogen-free conditions and were fed autoclaved food and reverse osmosis water. All protocols in this study followed guidelines provided by the Canadian Council for Animal Care and the University of British Columbia Animal Care Committee.

### *In Vivo* Experiments

#### Mammary Tumor Model

A total of 1 × 10^6^ MT or MT^GCSF^*^−^*^/−^ cells were injected subcutaneously into the left flank of randomized syngeneic female MMTV/*neu*^OTI/OTII^ or *Rag1^−^^/^^−^* host mice. All analysis was done with blinding. Tumor volume was calculated using calipers and the formula: (4/3)π(*rl* × *rw* × *rh*). Animals bearing tumors larger than 1.5 mm^3^ or ulcerated tumors were removed from the study. Blood was collected via cardiac puncture and kept in ethylenediaminetetraacetic acid (EDTA) or immediately treated with one-step Fix/Lyse solution (Thermo Fisher Scientific) for phosflow/CyTOF as we described recently (30). A fraction of blood was analyzed using a scil Vet abc hematology analyzer [Heska Canada Limited (Formerly scil animal care company Canada)]. After blood collection, mice were perfused using fixative solution (1X Fix/Lyse Solution eBioscience) and organs were harvested, fixed for 30–60 minutes, and processed for flow and/or mass cytometry. Fixed splenocytes were passed through a 70 μm sieve. Tumors were mechanically dissociated using gentleMACS C Tubes (Miltenyi Biotec), resuspended and washed in plain RPMI and digested using Collagenase type I and type XI, Hyaluronidase and DNase I at 37°C for 40 minutes. Cells were counted using a hemocytometer and processed for CyTOF as described below. Nonfixed tissues were harvested without perfusion and processed as above. For adoptive T-cell therapy, naïve CD8^+^ OT-I T cells were isolated using CD8α and anti-biotin magnetic beads (Miltenyi Biotec) and washed twice in sterile PBS prior to injection. From 1 to 5 × 10^6^, CD8^+^ T cells were injected intravenously into tumor-bearing mice. Each *in vivo* study contained at least four replicates and each study was repeated at least three times. GCSF production by MT or MT^GCSF−/−^ was assessed by cytometric bead array (CBA) for mouse GCSF, following the manufacturer's guidelines (BD).

#### Azoxymethane/Dextran Sulfate Sodium Model of Colorectal Cancer

Host mice received an intraperitoneal injection of azoxymethane (AOM; 10 mg/kg). 5 days later, mice received dextran sulfate sodium (DSS) at 2.5% *ad libitum* in drinking water for 7 days, followed by a second 5-day cycle at 2.5% and a third DSS cycle (5 days, 2.0%). DSS cycles were interspaced with a recovery cycle with sterile drinking water for 14 days. At the end of the third recovery cycle, mice were bled and received 25 μg of either isotype (IgG1, R&D Systems) or anti-GCSF (MAB414, R&D Systems) antibody intraperitoneally three times a week for 3 weeks. Colons were harvested, cleaned, washed and tumor load was assessed under a dissection microscope. Tissues were processed to a single-cell suspension as described previously (31). Each *in vivo* study contained at least four replicates and the study was repeated three times.

### *In Vitro* Experiments

Cell lines MT and OP9-DLL1 were provided by Dr. Brad Nelson and Dr. Juan Carlos Zuniga-Pflucker, respectively. Cell lines were tested (2018) for *Mycoplasma* using RADIL, and authentication was done by FACS analysis of surface marker expression. Cells were passaged <7 times after thawing.

#### DC Generation

Femurs were harvested in sterile conditions and flushed with DC media [DMEM (Sigma), 10% heat-inactivated FBS (Invitrogen), 100 U/mL penicillin-G and 100 μg/mL streptomycin (Thermo Fisher Scientific), 2 mmol/L glutaMAX (Invitrogen), 55 μmol/L B-mercaptoethanol]. Cells were pelleted, resuspended in fresh media, and counted using a hemocytometer. BM cells (10^6^ in 2 mL) were plated in a 24-well plate supplemented with Flt3 L (100 ng/mL) for 6 days (DC progenitor analyses) or 9–10 days (DC maturation and function assays). Three technical replicates together with three biological replicates were used per condition per study. Half of media was replaced by fresh media on day 6. Mammary tumor-conditioned medium (MT-CM) was produced by plating 5 × 10^6^ MT or MT^GCSF^*^−^*^/−^ cells in 25 mL of MT growth medium [RPMI1640, 10% heat-inactivated FBS (Invitrogen), 100 U/mL penicillin-G and 100 μg/mL streptomycin (Thermo Fisher Scientific), 2 mmol/L glutaMAX (Invitrogen), 55 μmol/L β-mercaptoethanol (Sigma-Aldrich) and 1x insulin/transferrin/selenium (Lonza)] in T175 flasks for 4 days. MT-CM was collected following centrifugation, aliquoted and stored at −80°C. These DC BM cultures were exposed to 5% of MT- or MT^GCSF−/−^-CM throughout the protocol. Control BM cultures received 5% MT growth medium. DLL1-DCs were grown as described previously (32).

To assess T-cell proliferation, DCs were first grown for 7 days, by culturing 10^6^ BM cells in 2 mL of either DC medium, or DC medium supplemented with 5% conditioned medium from MT cells. On day 3, cells were transferred onto an OP9DLL1 monolayer and cultured for an additional 4 days. On day 6, DC cultures were pulsed with chicken ovalbumin (OVA; Sigma-Aldrich, 100 μg/mL) for 24 hours. On day 7, OVA-pulsed DCs were washed, counted, and plated with naïve OT-1 CD8^+^ T cells in triplicate. OT-1 T cells were freshly isolated from the spleen of OT-1 mice using Miltenyi's CD8 enrichment kit, and subsequently labeled with violet cell tracker proliferation dye following the manufacturer's guidelines (Thermo Fisher Scientific). The final ratio of DC/OT-1 CD8^+^ T cells was 5:1 and cultures were incubated for 72 hours in T-cell medium [DMEM, 10% FBS, Pen/Strep (50 U/mL), 1x Pyruvate (1 μmol/L), Glutamax (2 mmol/L), 2-ME (50 μmol/L) 1X non-essential amino acids]. Cells were stained with antibodies recognizing CD8, CD3, and Thy1 to identify T-cell surface markers, and acquired using an LSR-II cytometer.

#### Colonic Cultures

Host mouse colons were harvested and washed three times in PBS containing penicillin (100 U/mL) and streptomycin (100 μg/mL), and 0.5 cm of the distal portion of the colon was cultured in DMEM + penicillin/streptomycin and gentamycin (100 μg/mL) at 37°C for 24 hours. Media was collected and stored at −80°C.

### Flow and Mass Cytometry Sample Preparation, Data Acquisition, and Analysis

For flow cytometry, cells were processed into a single-cell suspension in FACS buffer. Fc receptor blocking was performed using rat serum and dead cells were excluded using Fixable Viability Dye (flow, eBioscience) or cisplatin (CyTOF, DVS Sciences). After washing, 3 × 10^6^ cells were labeled with surface markers using direct fluorochrome conjugates or biotinylated antibodies, and a streptavidin-conjugated secondary antibody. For CyTOF, 3 × 10^6^ cells of each sample were permeabilized (Fluidigm Maxpar Fix and Perm Buffer) and barcoded (Fluidigm Cell-ID 20-Plex Pd Barcoding Kit) and subsequently pooled. Cells were then stained with mAbs specific to cell surface and signaling proteins. Cells were acquired using an LSR II flow cytometer or a CyTOF II. Data were analyzed using FlowJo analysis software (Tree Star) or Cytobank (www.cytobank.org). Red blood cells were excluded by gating on CD45^+^ cells and debris removed using a DNA intercalator. Antibodies were purchased from eBioscience, BD Biosciences, BioLegend, Fluidigm, and the Biomedical Research Centre [University of British Columbia (UBC)]. Purified antibodies were conjugated to lanthanides at the Biomedical Research Centre. *t*-stochastic neighbor embedded (tSNE) dimensionality reduction algorithm was used to visualize (viSNE) the CyTOF data (http://www.cytobank.org). Original files were randomly downsampled using Cytobank. Equal numbers of gated events were analyzed per condition to ensure each replicate had the same impact on the final tSNE coordinates. The maximum number of events combining all replicates and conditions of a single tissue was 100,000 events.

#### tSNE Parameters

iterations 1,000, perplexity 30, theta 0.5 (default at CytoBank). For FlowSOM analysis, the number of clusters was first determined using Phenograph and constant K determined by the software. FlowSOM was then applied to generate plots and clustering/heatmaps.

### Flow and Mass Cytometry Panels

For CyTOF, two panels were used (Supplementary Table S1). The panel design tool provided by Fluidigm (now Standard BioTools Inc.) was used. Noisy channels (e.g., 157 Gd) were unused to minimize signal spill over. Antibodies against bright/widely expressed antigens were in “dimmer” channels and antibodies against rare or low expressing proteins were used in channels with high sensitivity/less prone to spill over. The first panel with 42 markers was used on live/nonfixed samples and consisted of antibodies targeting proteins on the cell surface of immune cells. This panel was used on BM cells and included marker used to identify progenitor cells in this tissue. The second panel contained lineage antibodies and antibodies targeting intracellular proteins. In this case, samples were fixed. Markers that were not required for delineating major cell populations (CD150, CD24) or targets that did not withstand fixation (e.g., CD103) were removed to reduce background. Flow cytometry antibodies are listed in Supplementary Table S2.

### The Cancer Genome Atlas Data Analysis

We used batch-corrected GRCh37/hg19 RSEM RNA sequencing (RNA-seq) data and sample quality annotations from The Cancer Genome Atlas (TCGA), publicly available as PanCancer Atlas data resources (EBPlusPlusAdjustPANCAN_IlluminaHiSeq_RNASeqV2.geneExp.tsv and merged_sample_quality_annotations.tsv) from gdc.cancer.gov/node/977 (33). We obtained clinical outcome data from the PanCancer Atlas clinical information from gdc.cancer.gov/node/905 (34). For colorectal cancer, data were available for 597 primary tumor samples and 47 adjacent normal tissue samples that passed quality filters. Of the available 47 normal, 45 are histologically normal tumor-adjacent tissue from the same patient that has matched corresponding tumor data. Only two are unmatched (did not pass quality filters). To define the colorectal cancer consensus molecular subtypes (CMS), we used the CMSclassifier (rdrr.io/github/Sage-Bionetworks/CMSclassifier (35)) on log_2_(RSEM + 1)-transformed RNA-seq data for primary tumors.

### Statistical Analyses

Two-tailed, unpaired Student *t* tests with a 95% confidence interval were performed on graphs generated in GraphPad Prism. Error bars represent SEM. *P* values of <0.05, <0.01, and <0.001 were used as cutoffs for statistical significance and are represented in the figures by one, two, or three asterisks, respectively. CyTOF expression/signal differences were assessed using significance analysis of microarray (SAM) unpaired test using a FDR-adjusted q-value of 0.01. SAM was implemented in R. One-way ANOVA was used to assess significance elsewhere. For expression heatmaps, a min-max transformation was performed on a per-marker basis to highlight the differences across the various tSNEs clusters. For analysis of human TCGA data, we used Kruskal–Wallis and Dunn multiple comparisons tests to compare gene expression in colorectal cancer tumors and adjacent normal tissue, and evaluated Spearman correlations of gene expression within colorectal cancer tumor samples. Kaplan–Meier analyses were performed with GraphPad Prism version 8, using the log-rank test, and a median split to define low and high expression within tumor samples.

### MT GCSF Knockout (MT^GCSF−/−^) Cells

CRISPR vectors were constructed using the GeneArt CRISPR Nuclease (OFP Reporter) Vector Kit (Invitrogen). NOP12 cells were transfected with the CRISPR Nuclease Vector containing the insert AGGACGAGAGGCCGTTCCCC, using Lipofectamine 3000. Four days later, cells were sorted for OFP expression and single cells were plated by limiting dilution. Colonies were screened using a BD Cytometric Bead Array for GCSF (Beckton-Dickinson). Sanger sequencing revealed a loss-of-function (LOF) mutation in nucleotide 33 within exon 2 of the murine *csf3* gene.

### Data Availability

Data were generated by the authors and available on request.

## Results

### GCSF Regulates Tumor Immune Cell Composition and Signaling

We first characterized tumor stroma immune infiltrate in mice bearing the GCSF-secreting mammary adenocarcinoma cell line NOP12 (referred to here as MT). MT cells are derived from the MMTV-*neu* OT-I/OT-II mammary tumor mouse model. MT cells express activated rat HER2/*neu* tagged at its C-terminus with CD4 and CD8 T-cell epitopes from OVA, together with a dominant-negative p53 transgene (C57BL/6-MMTV*/neu*^OTI/OTII^; ref. 36). When injected subcutaneously into the flank of syngeneic mice, MT cells form solid tumors with pronounced vasculature. MT tumors exhibit incomplete responsiveness to adoptive cell therapy with transgenic OT-I CD8^+^ T cells (36).

To assess GCSF's role in tumor development and responses to adoptive T-cell therapy, a loss-of-function mutation was introduced into the *Csf3* gene (encoding GCSF) using CRISPR/Cas9 mutagenesis. Four MT clones lacking GCSF expression were identified by CBA and expression of surface markers including Neu, SIINFEKL/H2kb, and PD-L1 were similar between MT and MT^GCSF−/−^ cells (Supplementary Fig. S1A and S1B). We used clone #4 for the remaining experiments. MT^GCSF−/−^ cells grew similarly to MT cells following subcutaneous injection into syngeneic MMTV/neu^OTI/OTII^ or Rag1^−/−^ hosts (Supplementary Fig. S1C and S1D), up to days 25–32 when MT-bearing mice reached humane endpoint.

**FIGURE 1:**
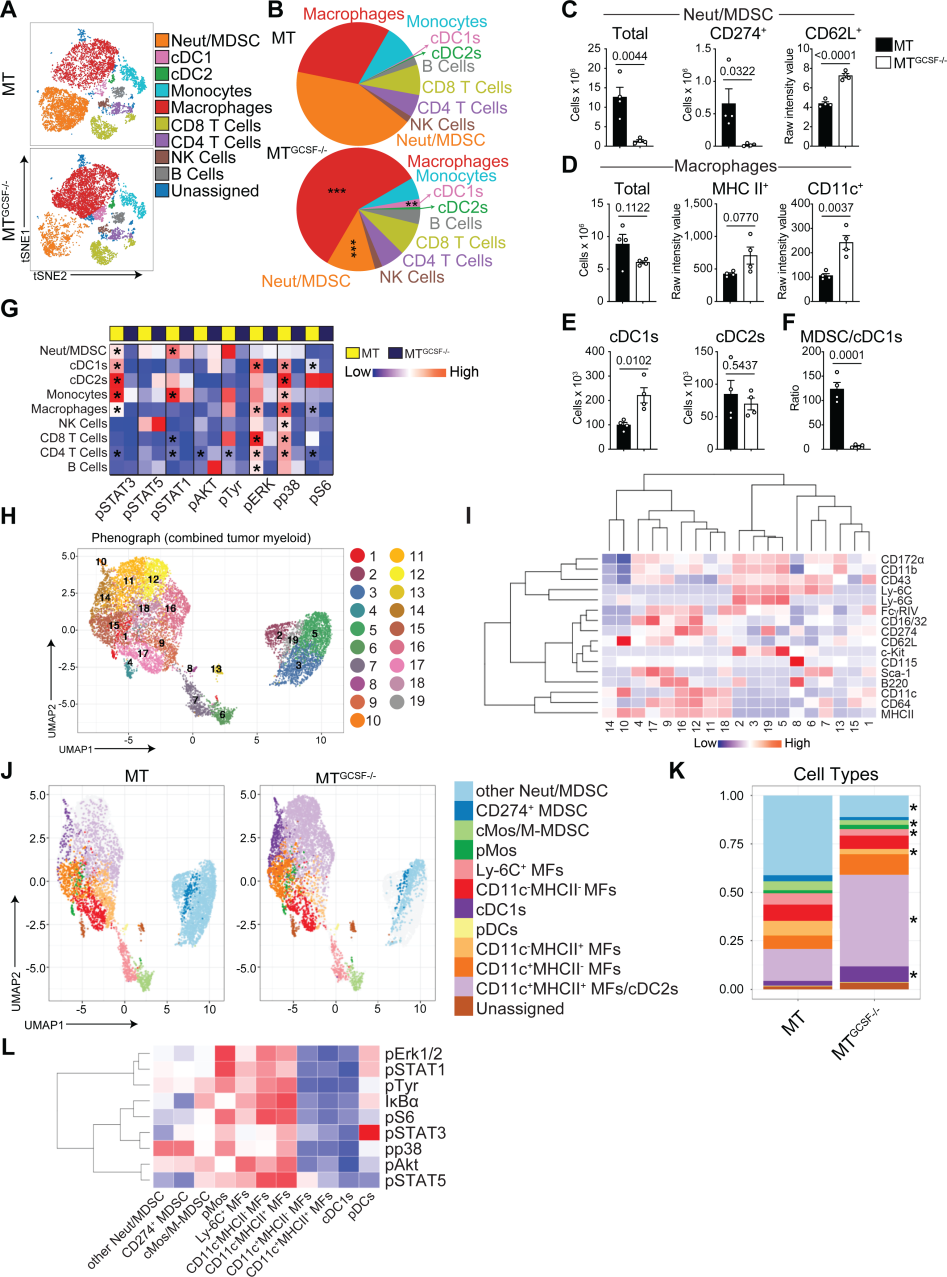
Tumor-derived GCSF alters tumor immune cell composition and signal transduction. Tumors from MT or MT^GCSF−/−^-bearing mice were digested, stained with up to 37 antibodies (surface panel) and 32 antibodies (phosphorylation panel), barcoded and assessed by CyTOF. Analysis of tumor stroma. **A,** Live CD45^+^ immune cells were gated and viSNE plots colored by sub gated populations. **B,** Relative frequency of subsets shown in A. **C,** Number of total Neut/MDSCs, PD-L1 (CD274)^+^ Neut/MDSCs per tumor, and CD62L^+^ expression by Neut/MDSCs. **D,** Total MF number per tumor, and expression of MHCII and CD11c. **E,** Number of CD103^+^ cDC1s and CD11b^+^ cDC2s per tumor. **F,** Ratio of Neut/MDSCs to CD103^+^ cDC1s. **G,** GCSF-dependent signaling changes assessed by CyTOF. The sample mean of the raw median marker expression value was used to generate heatmaps in R. **H–L**, To gate myeloid cells in an unbiased fashion, tSNE dimensionality reduction was run on all CD45^+^ cells from all samples. Next, myeloid cell subsets (i.e., cMos/M-MDSCs, Neuts/G-MDSCs, cDCs, pDCs, and MFs) were identified on the basis of surface protein expression patterns. **H,** Tumor-resident myeloid cells from MT or MT^GCSF−/−^ tumors were combined *in silico* and Phenograph was used for clustering and UMAP for dimensionality reduction. Myeloid cell subsets were annotated by their Phenograph clusters. **I,** Heatmap depicting the Z-score normalized expression levels for the indicated markers across the Phenograph clusters. **J,** Phenograph clusters were annotated on the UMAP plot as indicated cell subsets based on canonical surface marker expression patterns. **K,** Relative frequency of subsets shown in J. **L,** Heatmap depicting the Z-score normalized signal intensities for the indicated cell surface and intracellular phosphoproteins, respectively. Blue-white-red indicate lowest to highest signal intensity in heatmaps. Data were normalized (0–1 scale) per subpopulation Unpaired two-tailed Student *t* test was applied. Error bars represent SEM. *, *P* < 0.05; **, *P* < 0.01; ***, *P* < 0.001. Data represent two biological replicates, *n* ≥ 4 mice/group. MDSC refers to Neut/MDSC.

To determine the impact of tumor-derived GCSF on intratumoral and systemic immune cell populations and signaling profiles, euthanized mice were rapidly perfused with fixative to preserve *in vivo* signaling/phosphorylation. Single-cell suspensions of tumor, spleen, and BM were barcoded, pooled, and labeled with mAbs against 22 surface and nine intracellular markers (Supplementary Table S1) prior to mass cytometry (CyTOF; Supplementary Fig. S1F). Compared with MT^GCSF−/−^ tumors, GCSF-producing MT tumors contained an approximately 3-fold increase in total leukocyte numbers (CD45^+^ cells; Supplementary Fig. S1E) per tumor, dominated by Neut/MDSCs (42% vs. 12% of CD45^+^ cells). In GCSF-deficient tumors, cDCs and cDC1s were increased in frequency (∼3-fold) and there was an expanded proportion of TAMs. Proportions of CD4^+^ T cells, CD8^+^ T cells, B cells, and natural killer (NK) cells infiltrating tumors were not significantly changed by GCSF secretion (Fig. 1A and B).

Neut/MDSCs were increased approximately 10-fold within MT tumors, with a Neut/MDSC subset expressing PD-L1 (CD274) elevated approximately 30-fold. In addition, Neut/MDSCs from MT tumors exhibited reduced CD62 L levels (Fig. 1C), a phenotype associated with T-cell inhibition (37). This suggested that tumor-derived GCSF expanded tumor-resident MDSCs and altered their phenotype.

Although MT^GCSF−/−^ tumors showed an expanded proportion of TAMs, there was no change in numbers. However, TAMs in MT^GCSF−/−^ tumors expressed higher levels of CD11c and MHCII (Fig. 1D) and were characterized more thoroughly below.

cDCs, particularly CD103^+^ cDC1s, prime tumor-specific CD8^+^ T cells (38). cDC1s comprised less than 0.4% of CD45^+^ cells within the stroma of GCSF expressing MT tumors. In addition to the increased frequency of cDC1s in GCSF-deficient tumors noted above, there was an increase in their number (∼2-fold; Fig. 1E). Indeed, cDC1 were the only population to increase in numbers in MT^GCSF−/−^ tumors. Correspondingly, there was a significantly lower Neut/MDSC to cDC1 ratio in MT^GCSF−/−^ tumors (Fig. 1F). In contrast, no differences in CD11b^+^ cDC2s were observed between MT and MT^GCSF−/−^ tumors.

### Tumor-derived GCSF Activates STAT3, STAT1, and MAPK Pathways Within Tumor-infiltrating Immune Cells

Tumor-derived GCSF initiates signaling directly through GCSFR (JAK2-STAT3, PI3K-Akt, Ras-MAPK), or indirectly through secondary signaling cascades. CyTOF revealed that infiltrating myeloid cells like Neut/MDSCs, cDCs, Mos, and MFs in MT tumors exhibited strong pSTAT3 staining relative to those from MT^GCSF−/−^ tumors (Fig. 1G). There was a similar trend for Neut/MDSCs and Mos with respect to pSTAT1. Lack of GCSF also led to reduced pERK1/2 levels, particularly in B and T lymphocytes, TAMs, and cDC1s. Similarly, p38 activation was significantly reduced in the absence of GCSF (Fig. 1G). Therefore, tumor-derived GCSF led to widespread changes in signaling within tumor stroma immune cells.

### Phenograph Analysis of Intratumoral Phagocyte Subset Heterogeneity and Signaling

To characterize the impact of GCSF on phagocyte subset composition more closely, tumor CD45^+^ myeloid cells were subgated from all the samples. Myeloid cell subsets such as conventional monocytes (cMos), monocytic-MDSCs (M-MDSCs), Neut/granulocytic-MDSCs (G-MDSCs) cDCs, pDCs, and macrophages were identified on the basis of the surface marker expression patterns and combined *in silico* for analysis using Phenograph (39) clustering and uniform manifold approximation and projection (UMAP) dimensional reduction, revealing 19 distinguishable subsets based on cell-surface protein expression (Fig. 1H). Heatmaps showing Z-score–normalized signal intensities for each surface (Fig. 1I) are shown. The relative frequency of each grouped subset, manually annotated by cell-surface marker expression profiles, was calculated (Fig. 1J and K). MT^GCSF−/−^ tumors showed diminished Neut/MDSC frequencies including populations that express c-Kit, Ly6C, Ly6G, and CD11b (clusters 2, 3, 5, 19) and PDL1 (cluster 19) and therefore share key markers associated with an MDSC designation in tumors. There was also a relative increase in CD11c^+^MHCII^+/−^ cells. The relative frequency of cDC1s was also increased 3.6-fold amongst MPS cells in GCSF-deficient tumors. Phenograph analysis allowed interrogation of protein phosphorylation in subsets. Heatmaps showing Z-score–normalized signal intensities for each intracellular protein are shown (Fig. 1L). Interestingly, PD-L1(CD274^+^) Neut/MDSCs and CD11c^−^MHCII^+/−^ MFs displayed higher pSTAT3 and overall signaling intensity. Indeed, the expanded CD11c^+^MHCII^+/−^ MFs in GCSF-deficient tumors showed low phosphoprotein signal intensity. In this regard, CD11c^+^ MFs clusters that increased in MT^GCSF−/−^ tumors resembled cDC1s in terms of signaling profiles. In contrast, CD11c^−^ MFs and MDSCs (populations expanded in GCSF-expressing tumors) exhibited higher phosphosignaling signatures (Fig. 1L). GCSF therefore led to accumulation of tumor-resident Neut/MDSCs and CD11c^−^ TAMs with distinct phosphorylation/signaling signatures.

### Tumor-derived GCSF Induces Peripheral Changes in Immune Cells and Marked Granulocytic Expansion

We confirmed work from our lab and others (28, 39) showing that MT growth led to dysregulated hemopoiesis characterized by splenomegaly, granulocytic expansion, and anemia in both syngeneic MMTV and Rag1^−/−^ hosts (Supplementary Fig. S2). To assess the contribution of GCSF, we compared mice bearing MT and MT^GCSF−/−^ tumors. Mice lacking tumor-secreted GCSF resembled non–tumor-bearing mice with respect to counts of circulating white blood cells (WBC), granulocytes, and Mos, as well as spleen weights and BM red blood cells (Supplementary Fig. S2). Mice lacking the receptor for GCSF, but harboring GCSF-secreting MT tumors, had significantly lower WBCs, granulocytes, and spleen weights than did wild-type (WT) GCSFR expressing tumor-bearing mice (Supplementary Fig. S2C) confirming that these perturbations were GCSF/GCSFR dependent.

**FIGURE 2:**
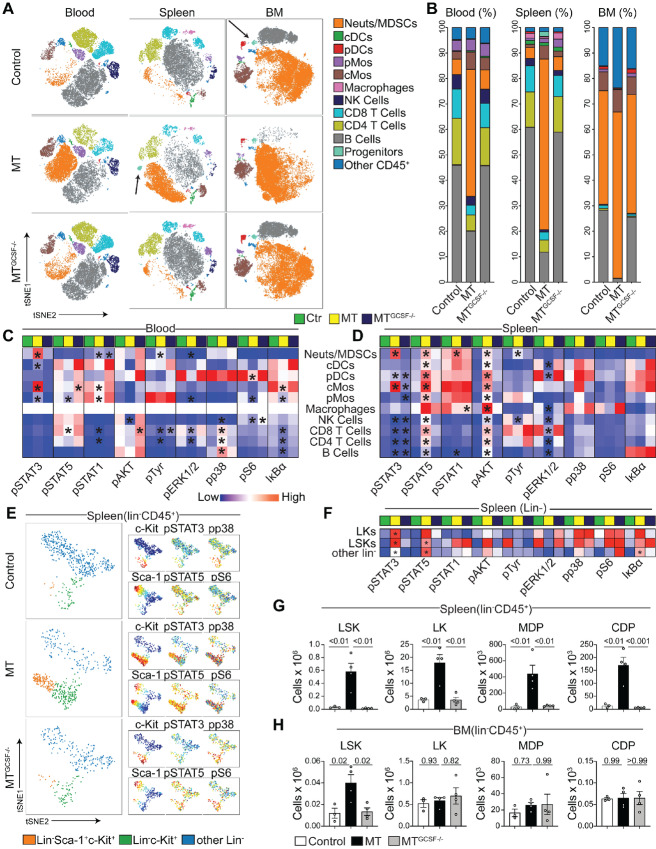
Tumor-derived GCSF alters blood, spleen, and BM immune cell composition and signal transduction. Blood, BM, and spleen from control, MT or MT^GCSF−/−^-bearing mice were stained with up to 37 antibodies and assessed by CyTOF. **A–D,** Analysis of blood, spleen, and BM. viSNE plots displaying immune cell subset distribution of indicated experimental groups (**A**) and their frequencies in each tissue (**B**). GCSF-dependent signaling changes in tumor-bearing mice assessed by CyTOF in blood (**C**) and spleen (**D**). **E,** viSNE (larger plots) of lin^−^ splenocytes from control, MT- and MT^GCSF−/−^-bearing mice reveal LKs and LSKs. Smaller plots show indicated marker expression. **F,** GCSF-dependent signaling changes in splenic HSPCs assessed by CyTOF. **G–H,** Counts refer to total cells/speen or BM. Quantification of LSKs, LKs, MDPs, and CDPs in the spleen (**G**) and BM (**H**) of Rag1^−/−^ mice. One-way ANOVA was applied. Error bars, SEM. Data represent two (A–F) or ≥3 (G, H) experiments. Blue-white-red indicate lowest to highest signal intensity in heatmaps. Arrows in A point to progenitors. Data were normalized 0–1 scale. Error bars, SEM. *, *P* < 0.05 compared with control. Data represent three experiments, *n* = 4.

Growth of MT but not MT^GCSF−/−^ tumors led to a significant increase in the CD11b^+^ Ly6G^+^ Neut/MDSC population in blood, spleen, and BM (Fig. 2A and B). While these cells comprised less than 10% of immune cells in blood and spleen of healthy mice, they expanded to 50% and 70% of cells in blood and spleen, respectively, of MT, but not MT^GCSF−/−^ mice. Similarly, in healthy BM, Neut/MDSCs comprised approximately 45% of cells; however, they increased to over 60% of MT but not MT^GCSF−/−^ BM. In BM, there was a shift in tSNE coordinates of the CD11b^+^ Ly6G^+^ Neut/MDSC population of MT compared with MT^GCSF−/−^ mice (Fig. 2A). To investigate this, FlowSOM was used identifying 15 subclusters within this Neut/MDSC population (Supplementary Fig. S3A and S3B). Some subclusters identified in healthy animals (clusters 0 and 1 in particular) were diminished in BM of MT but not MT^GCSF−/−^ hosts (Supplementary Fig. S3C). In contrast, subclusters 5, 6, 7, 9, 10, 13, and 14 were enriched in the femurs of MT-bearing mice and displayed lower expression of Ly6G and increased CD16/32, suggesting a more immature phenotype (ref. 40; Supplementary Fig. S3D). Subcluster 14, with higher expression of c-Kit (CD117), was rare in healthy and MT^GCSF−/−^-bearing mice but prominent in MT-bearing mice.

Compared with healthy mice, MT but not MT^GCSF−/−^ tumor growth was associated with significant reduction in blood and splenic B-cell frequency. Similarly, BM from healthy and MT^GCSF−/−^-bearing mice contained approximately 30% B lymphocytes while femurs of MT-bearing mice were almost devoid of B cells (Fig. 2A and B). Combining CyTOF with tSNE and FlowSOM, we identified different subsets of lineage^−^ CD19^+^ B cells in the BM. Most of these B-cell clusters were reduced in frequency in MT-bearing mice, compared with healthy or MT^GCSF−/−^-bearing mice (Supplementary Fig. S3E and S3F). Hierarchical clustering analysis showed that B-cell clusters 3, 5, 16, 17, 21, and 22, which are characterized by low MHCII and B220 (CD45R), were prominent in BM of MT hosts (Supplementary Fig. S3G). Proportions of CD4^+^ and CD8^+^ T lymphocytes were also reduced in spleen and blood of MT but not MT^GCSF−/−-^bearing mice (Fig. 2A and B).

Profiling phosphoprotein patterns in the blood and spleen of control, MT-, and MT^GCSF−/−^-bearing mice revealed that tumor-secreted GCSF led to a significant increase in pSTAT3 in cells associated with GCSFR expression, such as Neut/MDSCs and cMos (ref. 41; Fig. 2C and D). In spleen, pSTAT5 and pAKT tended to be increased in all populations, in a GCSF-dependent manner. In blood and spleen of MT-bearing mice, pERK1/2 tended to be decreased in all populations. Interestingly, in mice harboring GCSF-secreting tumors, pSTAT3 was significantly increased in DCs, B and T lymphocytes—cells not usually associated with GCSFR expression. GCSF expression is therefore associated with widespread changes in immune cell distribution and signaling profiles.

### Tumor-derived GCSF Modulates Expansion of DC-restricted Progenitors

We previously showed that MTs led to increased lineage negative, Sca1 positive, cKit positive cells (LSK), short term hemopoietic stem cells (ST HSC), and multipotent progenitors (MPP) in BM and spleen, and increased long term HSCs (LT HSC) and megakaryocytic erythroid progenitors (MEP) in the spleen (28). Here we used CyTOF to assess tumor-derived GCSF in this process, revealing an increase in hematopoietic stem/progenitor cells (HSPC) in spleen of MT, but not control or MT^GCSF−/−^-bearing mice (Fig. 2A, B, and E). HSPCs in spleen of MT mice showed intense pSTAT3 (Y705) tyrosine phosphorylation (Fig. 2F). Because of scarcity of these cells, we used flow cytometry to assess the impact of MT or MT^GCSF−/−^ tumors on progenitor cells in spleen and BM using Rag1^−/−^ mice, which lack B and T cells, and depleted other lineage^+^ cells using a cocktail of mAbs. We found a significant increase in the number of LSKs in the BM, and LSKs and lineage negative, ckit positive, Sca1 negative (LK) progenitors in the spleen of MT-bearing mice that was dependent on tumor-derived GCSF (Fig. 2G and H).

**FIGURE 3:**
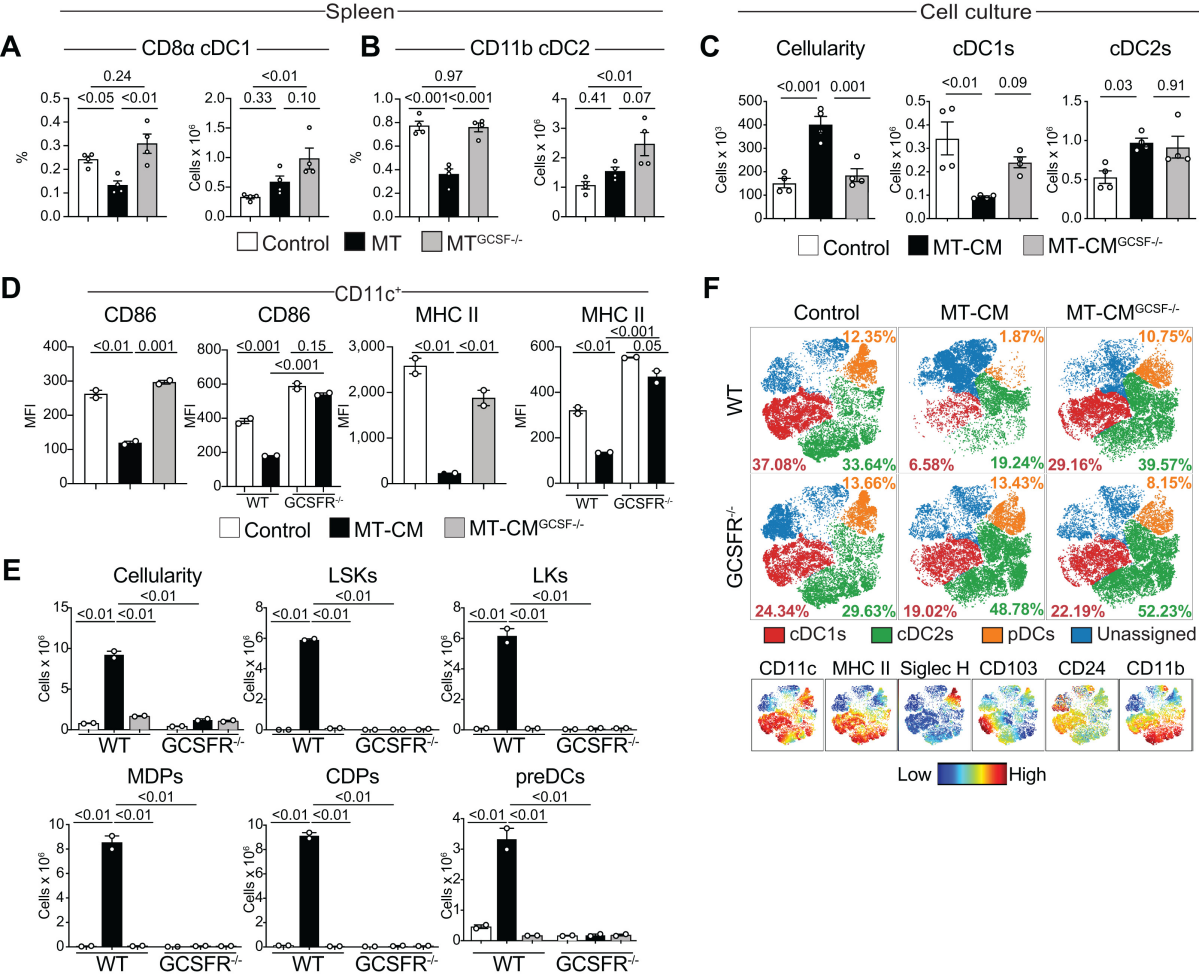
MT cell GCSF production impairs DC development and activation *in vitro* and *in vivo*. Proportions and numbers of cDC1s (**A**) and cDC2s (**B**) in the spleens of healthy, MT- and MT^GCSF−/−^-bearing mice. **C–F,** BM cells from WT or GCSFR knockout mice (GCSFR^-/−^) were cultured *in vitro* for up to 9 days in the presence of Flt3 L alone or with conditioned media from MT or MT^GCSF−/−^ cells and analyzed by flow cytometry. **C,** BM from WT mice was cultured as above. Quantification of total cells and cDC subsets on day 9. **D,** BM from WT or GCSFR^−/−^ mice was cultured as above. CD86 and MHCII MFI in CD11c^+^CD64^−^ cells on day 9. **E,** Proportions of cDC1s, cDC2s, and pDCs in cultures on day 9. Smaller plots show indicated marker expression. **F,** Quantification of LSKs, LKs, MDPs, CDPs, and preDCs in BM cultures on day 6. Lineage: CD3, CD19, B220, CD11b, MHCII, Ter119, Ly6G, Ly6C, DX5, NK1.1. One-way ANOVA was applied. Error bars, SEM. Data represent three experiments.

Given that mice harboring MTs showed a significant decrease in frequency and numbers of cDC1s within the stroma (Fig. 1A and B), we focused on elucidating the effects that tumors, and tumor-derived GCSF have on DC development. We found that macrophage-DC progenitor (MDP) cell and common DC progenitor (CDP) populations increased dramatically in the spleens of MT but not MT^GCSF−/−^ tumor-bearing mice, but did not change in the BM (Fig. 2G and H). Despite the large increase of MDPs and CDPs in the spleens of MT-bearing mice, the frequency of progeny, lymphoid-resident cDC1s (CD8α^+^) and cDC2s (CD11b^+^) was significantly reduced, and the numbers of cDC1s and cDC2s remained unchanged, suggesting that GCSF promoted the accumulation of splenic DC precursors with diminished differentiation potential (Fig. 3A and B; Supplementary Fig. S1H).

### DC Development is Impaired in Cell Cultures Treated with MT but not MT^GCSF−/−^ Tumor-conditioned Medium

To further investigate the impact of tumor-derived GCSF on the DC compartment, we utilized Flt3 L BM-derived DCs (42). We compared DC development in control cultures with cultures exposed to conditioned medium from mammary-tumor cells (MT-CM) or MT^GCSF−/−^ cells (MT^GCSF−/−^-CM). After 9 days, BM exposed to MT-CM exhibited a 2.5-fold increase in cell number with a pronounced reduction in numbers of mature CD103^+^ cDC1s, but not cDC2s, compared with MT^GCSF−/−^-CM treated or control cultures (Fig. 3C). In addition, MT-CM, but not MT^GCSF−/−^-CM, led to a significant reduction in CD86 and MHCII expression within the CD11c^+^ population (Fig. 3D). Cultures of BM from mice lacking the receptor for GCSF resembled control cultures, regardless of whether MT-CM was present (Fig. 3D; Supplementary Fig. S4A), confirming that inhibition of DC development was dependent on GCSF. The frequencies of cDCs (CD11c^+^MHCII^+^CD64^−^) and pDCs (CD11c^+^CD11b^−^SiglecH^+^) were also significantly reduced in cultures containing MT-CM but not MT^GCSF−/−^-CM (Fig. 3E; Supplementary Fig. S4A).

Many cells in MT-CM but not MT^GCSF−/−^-CM–treated cultures lacked common markers for myeloid cells. Flow cytometry revealed these cells to be DC progenitors, such as MDPs, CDPs, and preDCs (ref. 43; Fig. 3F). LKs and LSKs were also significantly increased in MT-CM–treated cultures. MDPs, CDPs, LKs, and LSKs developed to control levels in BM cultures from mice lacking the receptor for GCSF, even when grown in the presence of MT-CM, confirming the requirement for GCSF signaling (Fig. 3F). The *in vitro* cultures therefore modeled spleens of MT-bearing mice, suggesting that GCSF stimulates accumulation of DC progenitors impaired in their ability to differentiate into cDCs.

Recent reports highlight the importance of DLL1-NOTCH2 signaling in cDC1 development (32, 44) showing that cells cultured in the presence of DLL1 better resemble *bona fide* mouse cDCs. Using this method, we confirmed that cDCs expressed CD8α and DEC205, as expected. By adding MT- or MT^GCSF−/−^-CM to the cultures, we found that tumor-derived GCSF was equally potent at blocking cDC1s propagated with DLL1/NOTCH2 signaling (Supplementary Fig. S4B and S4C).

The ability of DCs propagated in the presence of tumor-conditioned medium ± GCSF to activate CD8^+^ T cells was then assessed. BM cultures were incubated with OVA, and OT-I CD8^+^ T-cell proliferation was assessed. Control cultures were efficient at presenting antigen and inducing CD8^+^ T-cell proliferation (Supplementary Fig. S4D) while DCs grown in the presence of MT-CM showed reduced T-cell proliferation, and DCs grown in the presence of MT^GCSF−/−^-CM showed intermediate T-cell proliferation (Supplementary Fig. S4E). Overall, our data provide further evidence that tumor-derived GCSF impairs DC development, leading to an impaired ability to induce T-cell proliferation.

### Tumor-derived GCSF Poses a Barrier to Adoptive T-cell Transfer Immunotherapy

**FIGURE 4:**
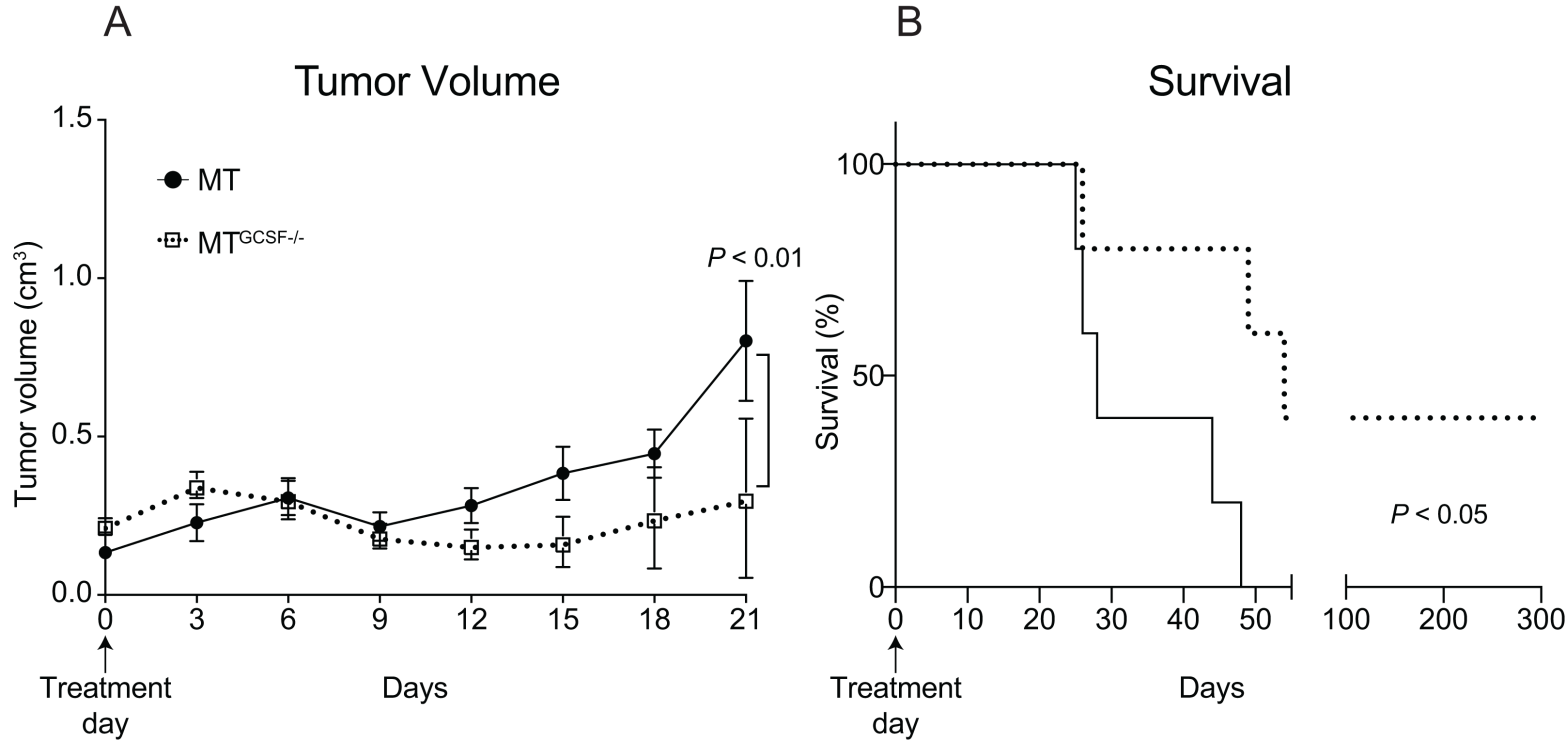
Tumor-derived GCSF poses a barrier to adoptive T-cell transfer immunotherapy. MMTV mice were transplanted subcutaneously with MT or MT^GCSF−/−^ cells. When tumors were approximately 0.3 mm^3^, mice (randomized) received 1 × 10^6^ naïve CD8 T cells from OT-I mouse donors. **A,** Tumor volume was assessed for 21 days posttreatment. The difference in volume between MT or MT^GCSF−/−^ tumors was not significant on days 0–18. **B,** Survival was monitored up to 300 days posttreatment. Error bars, SEM. Two-way ANOVA and Gehan–Breslow–Wilcoxon tests applied.

Our findings suggested GCSF inhibition could be of therapeutic value. To address this, mice bearing MT or MT^GCSF−/−^ tumors (∼30 mm^3^) were injected intravenously with naïve OT-I CD8^+^T cells, which recognize SIINFEKL/MHCI expressed on MT and MT^GCSF−/−^ tumor cells (Supplementary Fig. S4D and S4E; ref. 36). Tumor volumes were measured at 3-day intervals for 3 weeks after T-cell injection. We found that MT and MT^GCSF−/−^ tumor volumes were similar during the first week following adoptive T-cell transfer but by day 21, MT tumors were significantly larger than MT^GCSF−/−^ tumors (Fig. 4A). At 28 days posttreatment, MT-bearing mice approached a humane endpoint, and after 50 days, all MT-bearing mice were euthanized because of large tumor sizes. By contrast, MT^GCSF−/−^ tumors remained under control of the transferred CD8^+^ T cells, with significant improvement in survival. Importantly, 40% of MT^GCSF−/−^-bearing mice remained tumor-free for up to 300 days after treatment (Fig. 4B), suggesting that abrogation of tumor-derived GCSF improves adoptive T-cell therapy efficacy.

### AOM/DSS Colon Cancer Induces an Immunosuppressive Environment that Correlates with Tumor Load

**FIGURE 5:**
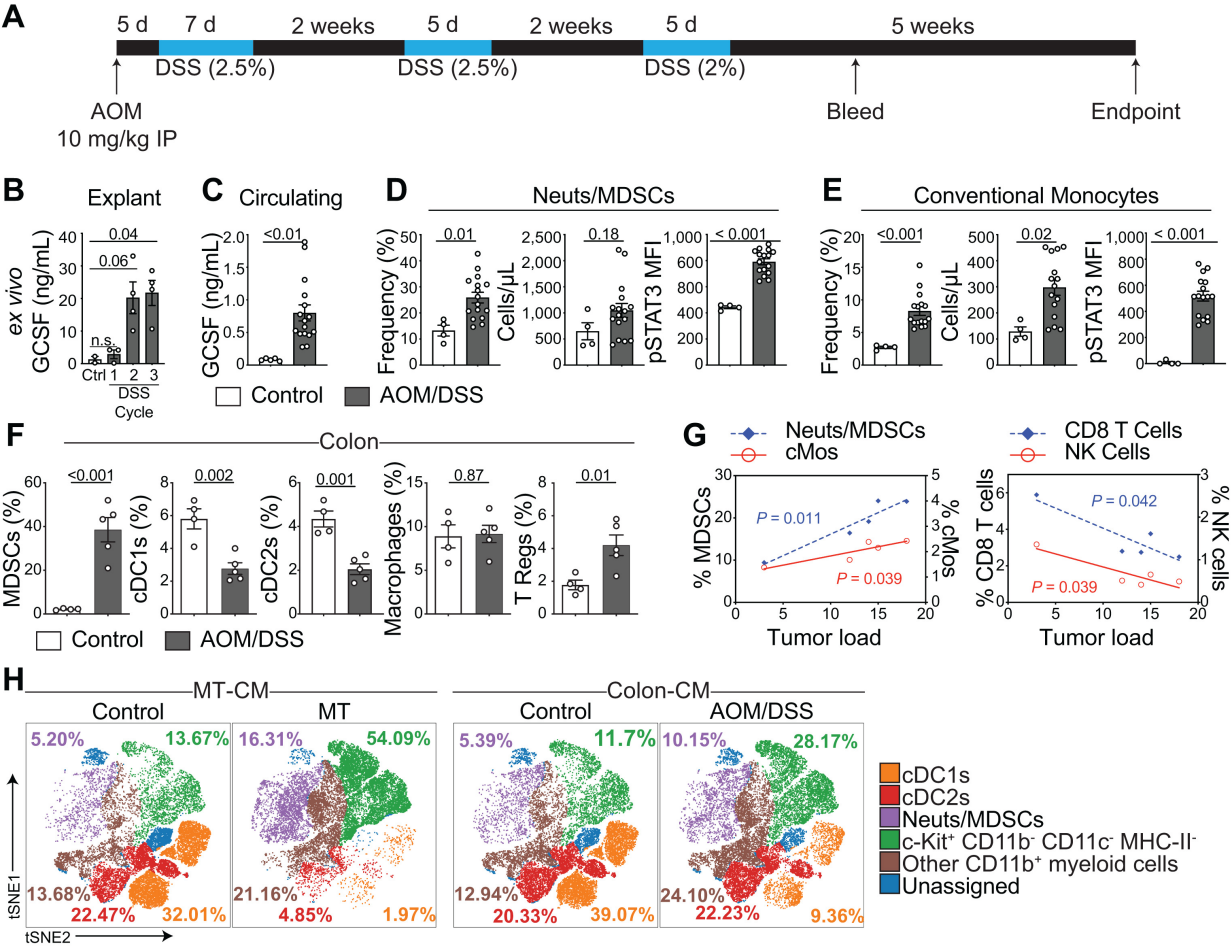
Tumor development in a spontaneous colorectal cancer mouse model leads to GCSF production and altered immune cell development and distribution. **A,** Diagram of the AOM/DSS-induced colorectal cancer model. **B,** Distal portion of healthy (Ctrl) or tumor-bearing colons were harvested 3 days after the indicated DSS cycle and incubated for 24 hours in plain media. GCSF was measured by CBA. **C,** Mice were bled 2 weeks after the third recovery cycle and circulating GCSF was assessed by CBA, as well as frequency, number (cells/μL) and pSTAT3 levels of Neut/MDSCs (**D**) and conventional Mos (**E**). **F,** CyTOF analysis showing frequency of indicated immune cell population in colons of healthy and tumor-bearing mice at endpoint (**G**) and correlations between immune cell subsets and tumor load at endpoint. **H,** Flt3 L cultures were incubated with MT^GCSF−/−^-CM, MT-CM (left), control colon-CM or colon-CM from tumor-bearing mice (right), showing that tumor-CM induced by AOM/DSS recapitulates the MT model. One-way ANOVA applied in B. Unpaired two-tailed Student *t* test applied in C–F. Simple linear regression was applied in G. Error bars, SEM. Data represent three experiments.

To further assess endogenously-upregulated GCSF in cancer, we utilized the mutagen-induced model of spontaneous colorectal cancer (AOM/DSS), where mice receive azoxymethane followed by three cycles of the inflammatory agent DSS, to induce neoplasms (Fig. 5A; ref. 29). Consistent with previous reports, colonic GCSF was detectable at DSS cycle 1 and 2, reaching statistical significance by cycle 3 (54 days; ref. 45; Fig. 5B). Circulating GCSF was also significantly increased after cycle 3 (Fig. 5C), along with an increase in the frequency of blood Neut/MDSCs and cMos, both of which exhibited elevated pSTAT3 (Fig. 5D and E). Tumor growth was associated with increased frequency of colonic Neut/MDSCs and Tregs and a decrease in cDC1s and cDC2s (Fig. 5F). In addition, tumor load at endpoint positively correlated with increased numbers of tumor-resident Neut/MDSCs and cMos, and was negatively correlated with CD8^+^ T-cell and NK-cell frequency (Fig. 5G).

As was the case with MT-CM, many tumor-induced myeloid cell changes could be recapitulated *in vitro*, using AOM/DSS colon-CM. Flow cytometry revealed that, when compared with healthy colon-CM, cultures exposed to AOM/DSS colon-CM contained a decrease in cDC1 frequency and an increase in the frequency of Neut/MDCSs and HSPCs (Lineage^−^ Kit^+^; Fig. 5H).

### GCSF Neutralization in the AOM/DSS Colon Cancer Model Restores Phagocyte Composition and Reduces Tumor Load

**FIGURE 6:**
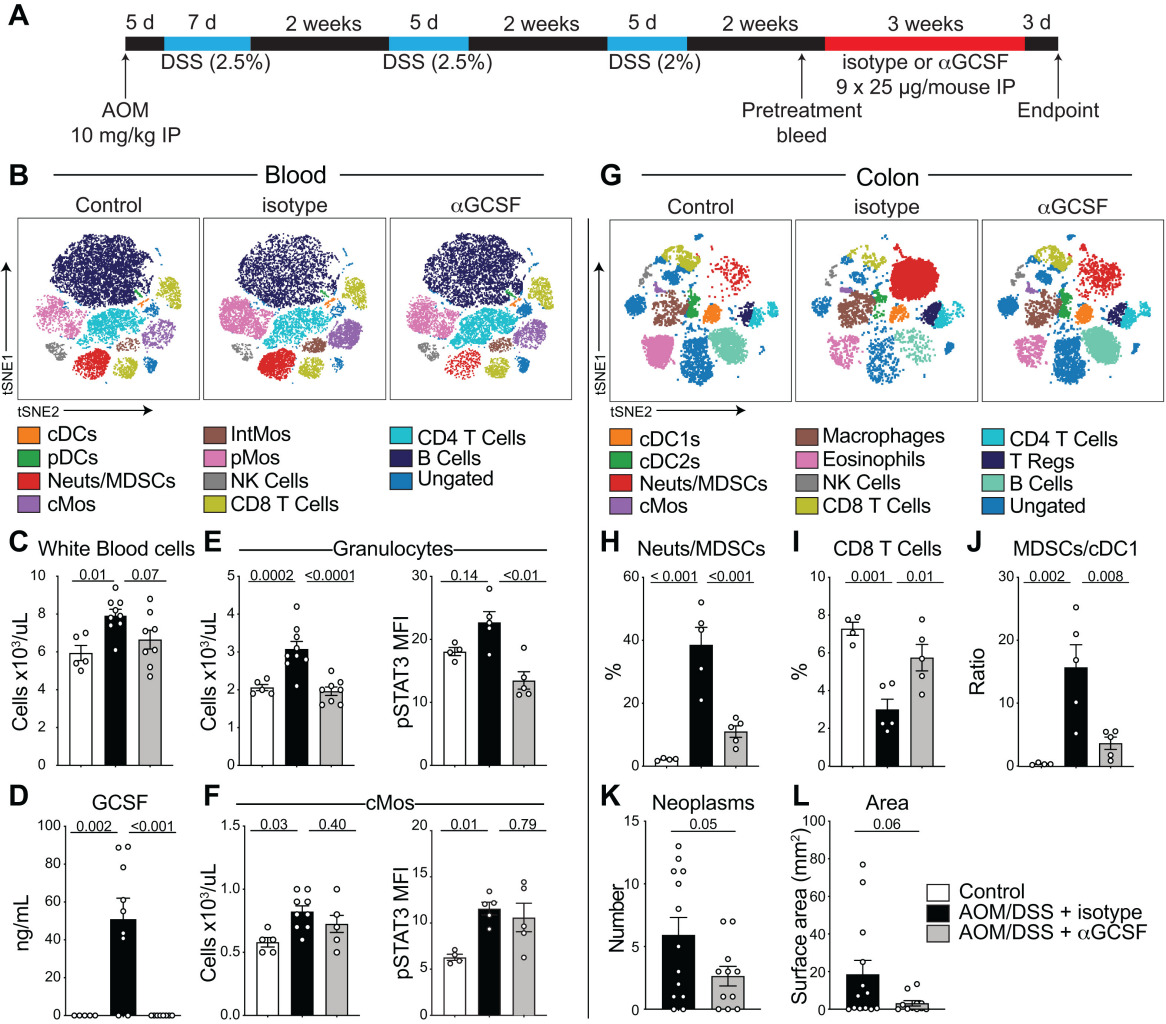
GCSF neutralization reduces Neut/MDSCs, increases CD8^+^ T cells, and leads to reduced tumor load. **A,** Diagram of AOM/DSS model and antibody treatment regimen. A total of 16 days after the third DSS cycle, mice received 25 μg MAB414 (anti-GCSF) or IgG1 isotype control i.p. three times a week for 3 weeks. **B,** CyTOF analyses of blood reveal changes in immune cell subset frequencies. **C,** WBC numbers at endpoint. **D,** Circulating GCSF at endpoint. Number and pSTAT3 MFI of Neut/MDSCs (**E**) and conventional Mos (**F**) at endpoint. **G,** CyTOF analyses of colonic tissue reveal changes in cell subset frequency. **H,** Colonic Neut/MDSCs. **I,** Colonic CD8^+^ T cells. **J,** Colonic Neut/MDSCs to CD103^+^ cDC1 ratio. Quantification of neoplasms (**K**) and neoplasm surface area (**L**) at endpoint. One-way ANOVA applied in C–F, H–J. Unpaired two-tailed Student *t* test applied in K–L. Error bars, SEM. Data represent two experiments.

To test the therapeutic benefit of neutralizing GCSF in the colorectal cancer model mice, AOM/DSS-treated mice were administered neutralizing anti-GCSF or isotype control (IgG1a) antibodies for 3 weeks, starting after recovery cycle 3 (Fig. 6A). Blood analyses revealed changes in immune cell composition (Fig. 6B) and circulating WBC numbers (Fig. 6C) consistent with GCSF expression being decreased with GCSF blockade. Indeed, we confirmed by endpoint CBA that GCSF was neutralized in the circulation of anti-GCSF treated but not isotype control-treated mice (Fig. 6D). In concordance with the MT model, anti-GCSF treatment reduced STAT3 phosphorylation in Neut/MDSCs (Fig. 6E). Anti–GCSF-treated mice exhibited a significant reduction in the number of circulating Neut/MDSCs. Importantly, GCSF neutralization did not induce neutropenia (Fig. 6E and F).

B cells were increased in anti–GCSF-treated colon tissue, and there was a trend toward increased cDC1, cDC2, and CD4^+^ T cells (Fig. 6G). As was the case for blood, Neut/MDSCs were significantly decreased, and CD8^+^ T-cell frequency was significantly increased, in anti–GCSF-treated colons (Fig. 6G and I) while Neut/MDSCs to cDC1 ratio was significantly decreased (Fig. 6J). Importantly, anti-GCSF treatment led to reduced neoplasm numbers and a trend toward reduced tumor surface area compared with the isotype control group (Fig. 6K and L) which correlated with normalization of Neut/MDSCs and other circulatory and colonic immune cell populations in tumor-bearing mice.

### GCSF is Elevated in Human Colorectal Cancer and is Linked to Poor Clinical Outcome

**FIGURE 7:**
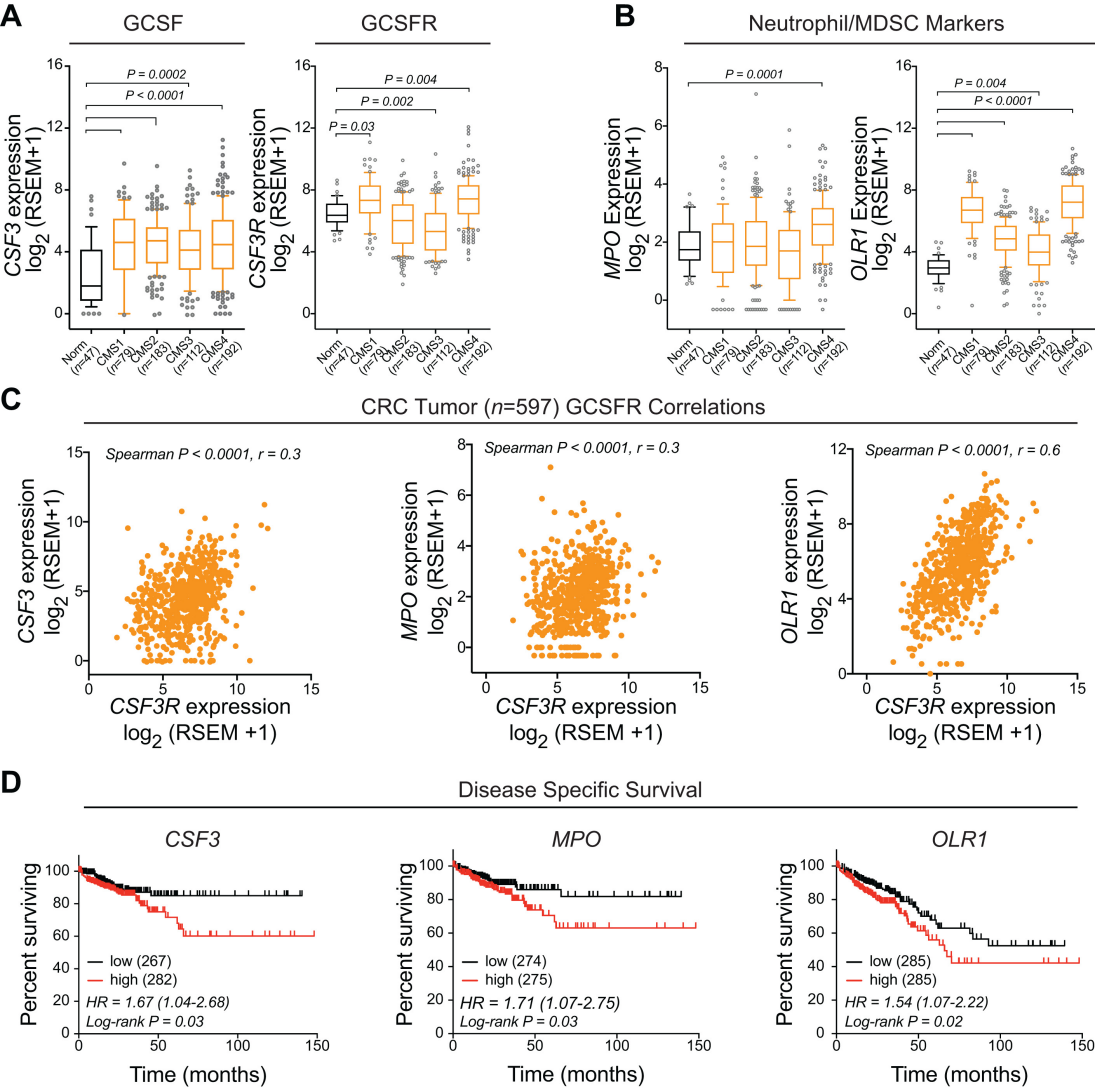
GCSF, GCSFR, and Neut/MDSC marker expression are associated with human colorectal cancer. Analysis of human colorectal cancer or adjacent normal tissue from TCGA data. Data were classified on the basis of consensus molecular subsets of colorectal cancer (CMS1–4). **A,** GCSF (*CSF3*) or GCSFR (*CSF3R*) expression. **B,** Expression of Neut/MDSC-associated genes, *MPO* or *OLR1*. **C,** GCSF (*CSF3*), *MPO*, and *OLR1* positively correlate with GCSFR (*CSF3R*) expression in human colorectal cancer. **D,** Median-split high versus low gene expression of GCSF (*CSF3*) or Neut/MDSC-associated genes *MPO* or *OLR1*, and survival outcome in human colorectal cancer. Kruskal–Wallis and Dunn multiple comparisons tests were applied in A and B. Tumor-only analysis was used in the Spearman correlation in C and the Kaplan–Meier log-rank test in D.

To investigate whether GCSF and Neut/MDSC gene expression are associated with human colorectal cancer, we analyzed 597 primary colorectal cancer tumor samples and 47 histologically normal tumor-adjacent tissue from the same patient that has matched corresponding tumor data, from TCGA database. We used a CMS classifier to identify the previously described colorectal cancer CMS (CMS1–4, *n* = 566; ref. 35). We found *CSF3* (GCSF) gene expression significantly elevated in all four colorectal cancer subtypes compared with adjacent normal tissue. *CSF3R* (GCSFR) expression is also more abundant in tumor tissue, specifically in subtypes CMS1, CMS3, and CMS4 (Fig. 7A). Correspondingly, the gene encoding myeloperoxidase (*MPO*), an enzyme highly expressed in human Neut, showed significantly elevated tumor expression in CMS4 (Fig. 7B). The gene encoding oxidized low-density lipoprotein receptor 1 (*OLR1*), a human granulocyte Neut/MDSC-specific marker (46) was significantly elevated in all molecular subtypes of colorectal cancer, with highest expression in CMS1 and CMS4 (Fig. 7B). These data support a greater Neut/MDSC infiltration in human colorectal cancer. Interestingly, *CSF3*, *MPO,* and *OLR1* gene expression positively correlated with *CSF3R* expression (Fig. 7C). Finally, based on a median low verses high gene expression split, high *CSF3*, *MPO,* and *OLR1* gene expression was associated with reduced disease-specific survival (*CSF3* and *MPO*) and overall survival (*ORL1*) in colorectal cancer (Fig. 7D).

## Discussion

Many human cancers exhibit high GCSF and/or GCSFR expression, including colorectal, breast, lung, ovarian, and pancreatic (23–27), and GCSF has been associated with tumor progression and poor therapy responses (24, 45, 47–50). Here we used murine breast and colon cancer models, together with a system-wide approach, to investigate the effects of tumor-derived GCSF on immune cell development and distribution, adoptive T-cell therapy, and tumor growth. Interestingly, tumor-associated GCSF led to remodeling of the immune cell content in tumor stroma, showing increased Neut/MDSCs at the expense of MFs and cDC1s. As well, spleen, blood, and BM of tumor-bearing mice became dominated by Neut/MDSCs and had diminished B cells, CD4^+^, and CD8^+^ T cell populations. This aligns with data from blood of patients with untreated breast cancer, which contains increased Neut/MDSCs and decreased Th1 and CD8^+^ T cells (51). We also showed that GCSF and Neut/MDSC-associated genes are elevated in human colorectal cancer, particularly the CMS1 and CMS4 subtypes, and are associated with poor outcome.

Neut/MDSCs are well characterized in terms of their ability to suppress immune responses in cancers and other inflammatory disorders, primarily by restricting activity of CD8^+^ T cells (10, 11) and NK cells, and recruiting Tregs (12). Using CyTOF, we found that GCSF led to elevated pSTAT3 in Neut/MDSCs in both cancer models, in keeping with the role of STAT3 in upregulating genes controlling Neut/MDSCs survival, expansion (52), and inhibitory functions (12). In agreement with others (51), we found that a subset of tumor-infiltrating Neut/MDSCs expressed high levels of PD-L1 suggesting a T-cell inhibitory function. Furthermore, deep phenotyping of the CD11b^+^ Ly6G^+^ Neut/MDSCs population in BM revealed that GCSF promoted an immature phenotype in multiple subpopulations. Effects of GCSF extended to tumor stroma, with GCSF-expressing MTs containing abundant pSTAT3 bright CD11c^−^MHCII^+/−^ subsets. In contrast, MTs lacking GCSF contained a higher frequency of CD11c^+^MHCII^+/−^ cells with lower pSTAT-1, -2, and -3 signatures. We contend that the GCSF-STAT3 axis plays a key role in regulating phagocyte heterogeneity and function within tumors. Our ongoing experiments are aimed at better distinguishing the subsets of MDSCs in tumors, blood, and organs (monocytic-MDSC, neutrophil vs. MDSC) using CyTOF and functional assays, and exploring the mechanistic balance between Neut and macrophages development, their progenitors and the impact of GCSF on this process (53). In contrast to Neut/MDSCs, cDC1s orchestrate antitumor immunity (19, 54–56), and make adoptive T-cell therapies more effective (57–61). We found that tumor-associated GCSF reduced the number of tumor-resident cDC1s in the breast cancer model, and in the colon cancer model there was a trend toward reduced cDC1s in colons of isotype versus anti-GCSF Ab-treated mice. In addition, these cDC1s showed heightened pSTAT3 activation, which is associated with suppression of DC maturation in both murine and human cancers (62–64).

To further determine the origin of expanded Neut/MDSC and contracted cDC populations, we examined the HSPC compartment, and confirmed the spleen is a niche for extramedullary hemopoiesis in MT-bearing mice, harboring elevated LSKs and LKs (28). We further identified an increase in DC precursors in the spleen of tumor-bearing mice, including MDPs and CDPs, as well as decreased cDC1s, all of which were dependent on tumor-secreted GCSF. We observed a similar dysregulation *in vitro*, using two different models of DC development from murine BM. Our findings align in several ways with those of Meyer and colleagues (65) who showed a GCSF-dependent reduction cDC1s in BM and tumors of human patients with cancer and mice. Mechanistically, enhanced STAT3 activity in DC precursors was associated with downregulation of IRF-8 (65), limiting Mo/DC differentiation and promoting granulocyte differentiation (66, 67). Taken together, we concluded that elevated GCSF led to a significant expansion of DC progenitors; however, these were deficient in their ability to differentiate into mature cDCs.

We found that tumor-secreted GCSF was necessary for reduction in B-cell numbers, in all compartments, in mammary cancer. This was particularly evident in BM, where B cells were almost absent with many B-cell subpopulations characterized by low maturation state. Similarly, in a colon cancer model, GCSF neutralization led to a significant increase in B cells within tumor-burdened colons. Others have detected reduced BM B cells in PyMT mammary tumor-bearing mice, but found that this was not likely a direct consequence of GCSF on B cells or their precursors, as antibody blockade of GCSF did not rescue B-cell numbers in an *in vitro* assay (68).

CD8^+^ T cells, a population capable of cytotoxic activity against tumor cells, were diminished in a GCSF-dependent manner in both breast and colon models of cancer, and in the latter, there was a significant inverse correlation between colonic CD8^+^ cytotoxic T cells and tumor load. We used CyTOF to assess tumor-resident T cells in tumors ± GCSF secretion and found that virtually all signaling was reduced in the absence of tumor GCSF, with a significant decrease in pSTAT5 in both CD4^+^ and CD8^+^ T cells, and pSTAT3 in CD4^+^ T cells. While both STAT5 and STAT3 activation can have a positive or detrimental effect, depending on the T-cell subset targeted (69), STAT3 activation is generally associated with a deficient antitumor immune response (70). Together, our data suggest that fewer and less-effective T cells are present in a GCSF^+^ tumor environment.

Given the dysfunctional immune environment that develops in the presence of GCSF, we and others (39, 45, 65, 71) postulate that GCSF inhibition would have therapeutic benefit. We found that colorectal cancer mice treated with neutralizing anti-GCSF Abs showed reduced Neut/MDSCs in multiple compartments, and an increase in colon-resident B cells and CD8^+^ T cells, suggesting a favorable immune environment. Importantly, we showed that anti–GCSF-treated mice had an approximately 58% decrease in tumor number, consistent with others who found a significantly lower number and size of tumors in anti–GCSF-treated mice (45). In the breast cancer model, we found that mice with GCSF^−/−^ tumors responded better than those with GCSF-expressing tumors to injection of tumor-reactive OT-I T cells, developing smaller tumors and exhibiting increased survival. Interestingly, a study by Allen and colleagues utilizing adoptive transfer of tumor experienced T cells, suggests that APCs, and not T cells themselves, are primarily responsible for T-cell dysfunction against bacterial infection in tumor-bearing mice (51); however, this remains to be investigated for T-cell dysfunction against tumors.

Although GCSF is best known for its use in the clinic to mobilize HSPCs in blood donors or to treat neutropenia after chemotherapy or radiotherapy, it is becoming apparent that GCSF associated with tumor growth can enhance tumorigenesis. Our data suggest that modulation of GCSF signaling in tumor-bearing mice diminishes aberrant STAT3-induced development and activation of immunosuppressive myeloid cells and boosts the frequency of immune-stimulatory cells such as cDC1s and CD11c^+^/MHCII^+^ MFs. GCSF inhibition also normalizes other hemopoietic parameters, including anemia, splenomegaly, and increased WBCs. We therefore contend that in cancers associated with production of GCSF, normalization of GCSF levels or GCSFR signaling may enhance antitumor immunity either alone or in conjunction with immune checkpoint blockade or adoptive T-cell therapy.

## Supplementary Material

Table TS1 CyTOF panels

Table TS2 Flow cytometry antibodies

Figure S1 Supplementary Figure S1 shows the characterization of MT and MTG-CSF-/- cell lines and tumors, including surface protein expression, tumor volume/weight, and immune cell subset content. Composition of immune cells in blood and spleen of MT and MTG-CSF-/- mice is also shown.

Figure S2 Supplementary Figure S2 compares the immune cell content in peripheral blood and spleens from immunocompetent, immunocompromised and G-CSFR-/- mice harboring MT or MTG-CSF-/- tumors.

Figure S3 Supplementary Figure S3 shows deep phenotyping of Neut/MDSCs and B cells in the bone marrow of mice bearing MT or MTG-CSF-/- tumors, using FlowSOM applied in tSNE maps.

Figure S4 Supplementary Figure S4 shows that G-CSF and G-CSFR signaling inhibit DC development and activation, in DCs derived in a OP9-DLL1 co-culture system.
