# Supplementary material for: Tumor-derived GCSF Alters Tumor and Systemic Immune System Cell Subset Composition and Signaling
Source: Cancer Res Commun. 2023 Mar 9;3(3):404–19. doi: 10.1158/2767-9764.CRC-22-0278 (PMC9997410; doi:10.1158/2767-9764.CRC-22-0278)
Supplement: Table TS1 — CyTOF panels [file crc-22-0278-s01.pdf]

Table S1. List of CyTOF antibodies used

	MT Model		AOM Model				
Lanthanide	Complete Panel (No Phosflow) (BM only)	Complete Panel (Phosflow)	Complete Panel (Phosflow)	Marker	Clone	Antibody Vendor	Catalog #
			CD45	CD45 (PAN)	30-F11	Fluidigm	3089005B
115Ln	CD11b	CD11b	CD11b	CD11b	M1/70	Biolegend	101249
			IL6	IL-6	MP5-20F3	UBC Antibody Lab	AB0000920
141Pr	MHCII	MHCII	MHCII	MHC-II	M5/144.15.2	Biolegend	107637
142Nd	B220	B220	CD279	CD279	29F.1A12	UBC Antibody Lab	AB0000820
143Nd	CD19		B220	B220	RA36B2	UBC Antibody Lab	21-0030-01
144Nd	CD206	pTyr	pTyr	p-Tyr	3144003A	Fluidigm	p-Tyr100
145Nd	cKit	cKit	FoxP3	FoxP3	NRRF-30	UBC Antibody Lab	AB0000820
146Nd	CD16/32	CD16/32	CD16/32	CD16/32	93	Biolegend	101335
147Sm	CD45 (pan)	CD45 (pan)	CD206	CD206	C068C2	UBC Antibody Lab	AB0000820
148Nd	CD24		Arginase-1	Arginase-1	polyclonal	UBC Antibody Lab	AB0000820
149Sm	CD103	CD103	CD103	CD103	2:E7	Biolegend	121401
150Nd	Flt3	STAT5	pSTAT5	pStat5[Y694]	47	Fluidigm	3150005A
151Eu	CD172a	CD172a	CD172a	CD172α	P84	Biolegend	144001
152Sm	CD90	pAKT	pAkt	pAkt [S473]	D9E	Fluidigm	3152005A
153Eu	CD44	STAT1	pSTAT1	pStat1[Y701]	58D6	Fluidigm	3153003A
154Sm	CD34		CD274	CD274	MIH5	UBC Antibody Lab	AB0000820
155Gd	NK1.1		NK1.1	NK1.1	PK136	UBC Antibody Lab	21-0056-01
156Gd	CD4	p38	pp38	pp38[T180/Y182]	D3F9	Fluidigm	3156002A
157Gd							
158Gd	SiglecH	STAT3	pSTAT3	pStat3[Y705]	4/P-STAT3	Fluidigm	3158005A
159Tb	CD43	CD43	CD43	CD43/SiglecH	eBioR2/60	ThermoFisher	14-0431-82
160Gd	CD115	CD115	CD115	CD115	AFS98	Biolegend	135521
161Dy	CD274	CD274	iNOS	CD274	MIH5	ThermoFisher	14-5982-82
162Dy	Sca1	Sca1	TNF-a	Sca1	D7	Biolegend	108135
163Dy	CD279		FcgRIV				
164Dy	F4/80	IkBa	IkBa	IkBα	L35A5	Fluidigm	3164004A
165Ho	CD150		IFN-g				
166Dy	CD11c	CD11c	CD11c	CD11c	N418	UBC Antibody Lab	21-0051-01
167Er	TCRB	TCRB	TCRb	TCRB	H57-597	Biolegend	109235
168Er	Ly6G	Ly6G	Ly6G	Ly-6G	1A8	UBC Antibody Lab	21-0066-01
169Tm	Ly6C (HK1.4)	Ly6C (HK1.4)	Ly6C	Ly-6C	HK1.4	Biolegend	128039
170Er	CD62L	CD62L	anti-biotin	CD62L	MEL-14	Biolegend	104443
171Yb	CD3	pERK1/2	pErk	pERK1/2[T202/Y204]	D13.14.4E	Fluidigm	
172Yb	CD8	CD8	CD8	CD8	53-6.7	UBC Antibody Lab	21-0012-01
173Yb	CCR7	CCR7	cKit	CCR7	4B12	Biolegend	120101
174Yb	CD64	CD64	CD64	CD64	X54-5/7.1	Biolegend	139301
175Lu	CD71	pS6	pS6	pS6 [S235/S236]	N7-548	Fluidigm	3175009A
176Yb	FcgRIV-APC	FcgRIV-APC	EpCAM (1/100) anti-APC(1/50)	FcγRIV/APC	9E9/APC 003	Biolegend/Fluidigm	149506/3176007B
209Bi	Ter119	Ter119	CD90.2	Ter119	Ter119	UBC Antibody Lab	21-0031-01
