# Supplementary material for: Tumor-derived GCSF Alters Tumor and Systemic Immune System Cell Subset Composition and Signaling
Source: Cancer Res Commun. 2023 Mar 9;3(3):404–19. doi: 10.1158/2767-9764.CRC-22-0278 (PMC9997410; doi:10.1158/2767-9764.CRC-22-0278)
Supplement: Table TS2 — Flow cytometry antibodies [file crc-22-0278-s02.pdf]

Table S2. List of flow cytometry antibodies used

Target Species	Marker	Clone	Antibody Vendor	Catalog #
Mouse	Phospho-STAT3 [Tyr705]	LUVNKLA	ThermoFisher	17-9033-42
Mouse	CD45	30-F11	ThermoFisher	48-0451-82
			Invitrogen	25-0451-82
			Invitrogen	17-0451-83
Mouse	TCR V alpha 2	B20.1	ThermoFisher	12-5812-82
Mouse	CD8α	53-6.7	BD Biosciences	553029
			BD Pharmigen	552877
			BD Pharmigen	557654
Mouse	CD11b	M1/79	Invitrogen	63-0112-82
			Invitrogen	53-0112-80
Mouse	MHC-II	I-A/I-E	Invitrogen	13-5321-81
			Invitrogen	17-5321-81
Mouse	Streptavidin		Invitrogen	46-4317-82
Mouse	CD64	X54-5/7.1	Biolegend	139304
			Biolegend	139315
Mouse	XCR1	ZET	Biolegend	148212
			Biolegend	148216
Mouse	CD117 (c-Kit)	2B8	eBioscience	17-1171-82
		ACK2	eBioscience	25-11/2-82
Mouse	Siglec H	eBio440c	Invitrogen	11-0333-82
Mouse	CD86	GL1	eBioscience	13-0862-81
Mouse	CD80	B7-1	eBioscience	17-0801-81
Mouse	CD135(Flt3)	A2F10	eBioscience	13-1351-82
Mouse	CD19	1D3	AbLab UBC	31-0055-01
Mouse	CD11c	N418	eBioscience	13-0114-82
			eBioscience	25-0114-81
			Invitrogen	47-0114-80
			AbLab UBC	31-0056-05
Mouse	NK1.1	PK136	eBioscience	13-5971-85
Mouse	CD49b	DX5	BD Biosciences	551460
			eBioscience	48-9668-82
			BD Biosciences	560600
			eBiosciences	31-0066-01
			Invitrogen	46-1721-80
Mouse	CD172	P84	eBioscience	45-5981-82
Mouse	Sca-1	D7	Invitrogen	17-1152-82
Mouse	CD115	AFS98	eBioscience	17-2051-80
Mouse	CD205	205yekta	Invitrogen	12-5961-81
Mouse	TCR-b	H57-597	Invitrogen	11-5961-82
