# Supplementary material for: Tumor-derived GCSF Alters Tumor and Systemic Immune System Cell Subset Composition and Signaling
Source: Cancer Res Commun. 2023 Mar 9;3(3):404–19. doi: 10.1158/2767-9764.CRC-22-0278 (PMC9997410; doi:10.1158/2767-9764.CRC-22-0278)
Supplement: Figure S1 — Supplementary Figure S1 shows the characterization of MT and MTG-CSF-/- cell lines and tumors, including surface protein expression, tumor volume/weight, and immune cell subset content. Composition of immune cells in blood and spleen of MT and MTG-CSF-/- mice is also shown. [file crc-22-0278-s03.pdf]

Figure S1

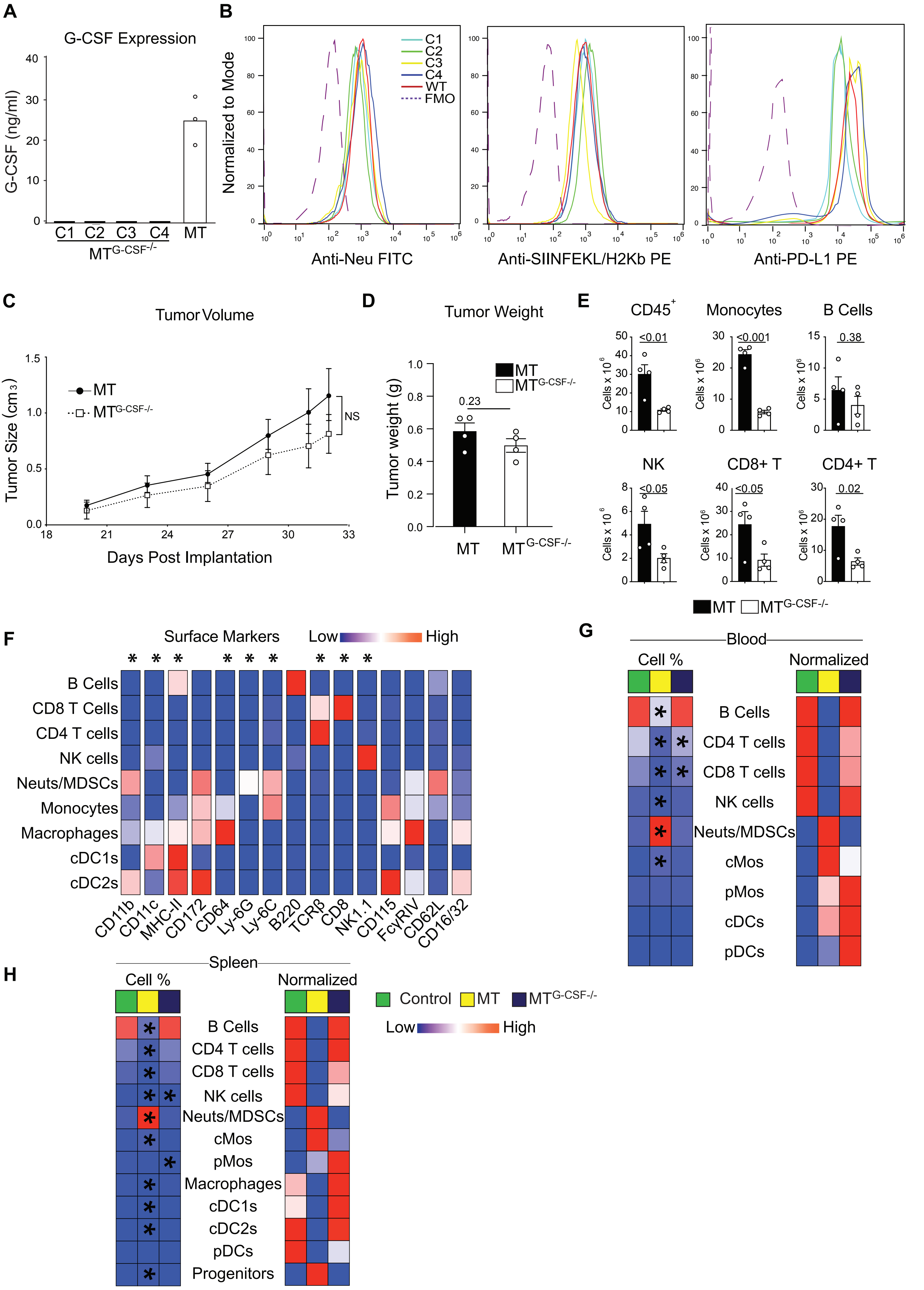
A
G-CSF Expression
G-CSF (ng/ml)
C1 C2 C3 C4 MT
MT^G-CSF-/-
B
Normalized to Mode
C1
C2
C3
C4
WT
FMO
Anti-Neu FITC
Anti-SIINFEKL/H2Kb PE
Anti-PD-L1 PE
C
Tumor Volume
Tumor Size (cm3)
MT
MT^G-CSF-/-
NS
Days Post Implantation
D
Tumor Weight
Tumor weight (g)
MT
MT^G-CSF-/-
0.23
E
CD45+
Monocytes
B Cells
NK
CD8+ T
CD4+ T
Cells x 10^6
≤0.01
≤0.001
0.38
≤0.05
≤0.05
0.02
MT MT^G-CSF-/-
F
Surface Markers
Low High
B Cells
CD8 T Cells
CD4 T cells
NK cells
Neuts/MDSCs
Monocytes
Macrophages
cDC1s
cDC2s
CD11b CD11c MHC-II CD172 CD64 Ly-6G Ly-6C B220 TCRβ CD8 NK1.1 CD115 FcγRIV CD62L CD16/32
G
Blood
Cell %
Normalized
B Cells
CD4 T cells
CD8 T cells
NK cells
Neuts/MDSCs
cMos
pMos
cDCs
pDCs
H
Spleen
Cell %
Normalized
B Cells
CD4 T cells
CD8 T cells
NK cells
Neuts/MDSCs
cMos
pMos
Macrophages
cDC1s
cDC2s
pDCs
Progenitors
Control MT MT^G-CSF-/-
Low High

Figure S1. Characterization of MT and MT$^{G\text{-}CSF-/-}$ cells and tumor-bearing mice. (A) CBA analysis of secreted G-CSF (conditioned medium) from parental MT cells, and four MT$^{G\text{-}CSF-/-}$ clones. (B) Surface expression of HER2/Neu, SIINFEKL/H2Kb and PD-L1 on parental MT cells and four MT$^{G\text{-}CSF-/-}$clones. For remaining experiments clone 4 was used. (C) Tumor volume of MT and MT$^{G\text{-}CSF-/-}$ tumors in MMTV mice (p=0.54). (D) MT and MT$^{G\text{-}CSF-/-}$ tumor weight at endpoint in Rag1$^{-/-}$ hosts. (E) Total numbers of tumor leukocytes, conventional monocytes, B cells, NK, CD8^{+} T and CD4^{+} T cells per tumor. (F) Heatmap showing guide to delineate populations. Asterisks indicate markers used for clustering in Fig. 1A. (G) Proportions of the indicated immune cells in blood and (H) spleen. For G and H, data was normalized (0-1 scale) per subpopulation (row) in the heatmap on the right. Blue-white-red indicate lowest to highest signal intensity in the heatmaps. A two-way ANOVA was used in C, and a one-way ANOVA in G-H. Unpaired two-tailed Student's t-test applied elsewhere. Error bars represent SEM.
