# Supplementary material for: Tumor-derived GCSF Alters Tumor and Systemic Immune System Cell Subset Composition and Signaling
Source: Cancer Res Commun. 2023 Mar 9;3(3):404–19. doi: 10.1158/2767-9764.CRC-22-0278 (PMC9997410; doi:10.1158/2767-9764.CRC-22-0278)
Supplement: Figure S2 — Supplementary Figure S2 compares the immune cell content in peripheral blood and spleens from immunocompetent, immunocompromised and G-CSFR-/- mice harboring MT or MTG-CSF-/- tumors. [file crc-22-0278-s04.pdf]

Figure S2

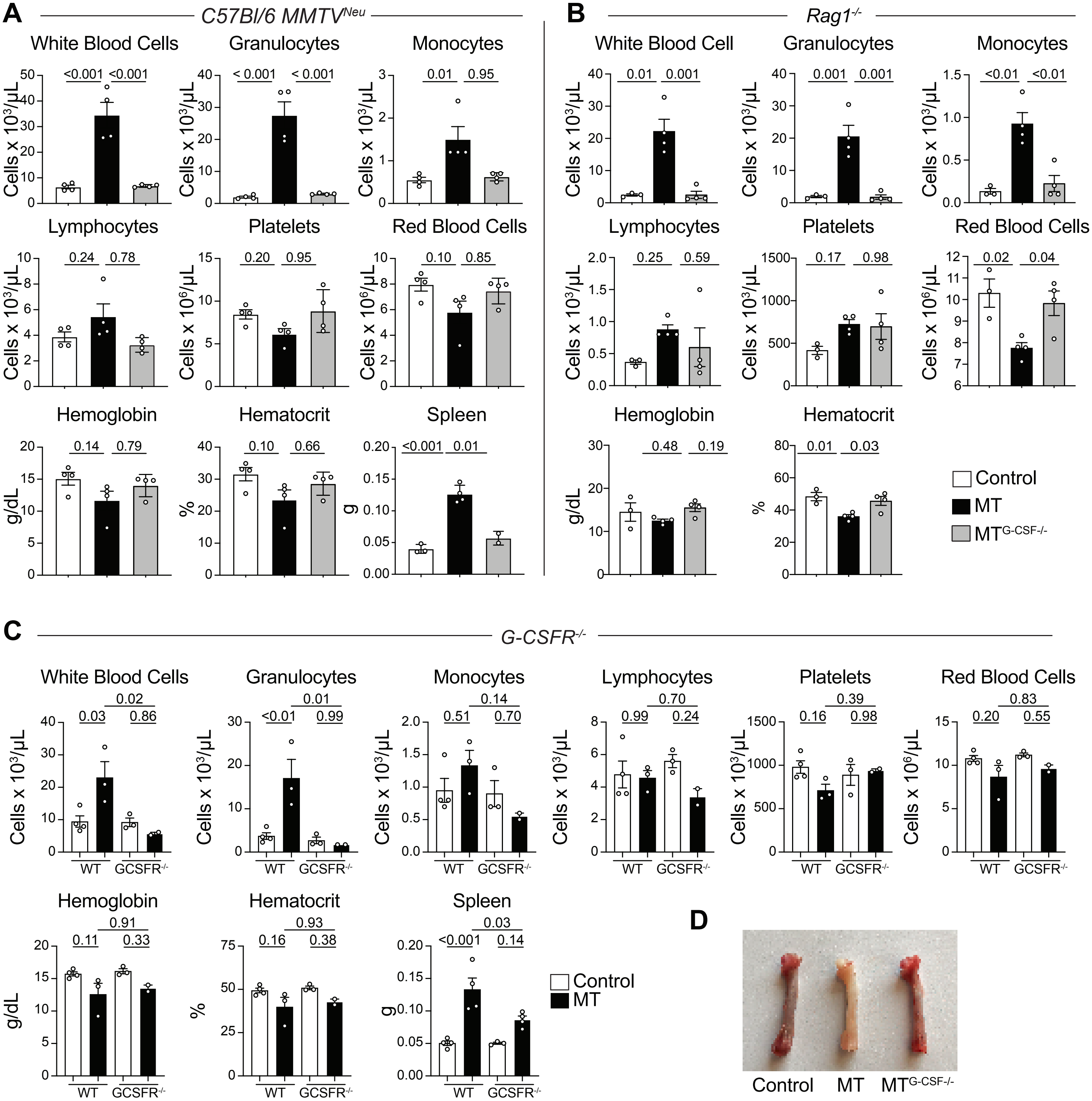

Figure S2. **Peripheral blood and spleen weights of immunocompetent, immunocompromised and G-CSFR$^{-/-}$ mice harboring MT or MT$^{G-CSF-/-}$ tumors.** Blood of control, MT- or MT$^{G-CSF-/-}$-bearing mice was collected and analyzed using a Vet abc blood analyzer. Blood parameters of (A) MMTV, (B) Rag1$^{-/-}$ and (C) MMTV and MMTV/G-CSFR$^{-/-}$ mice are shown, along with spleen weight of healthy and MT-bearing MMTV and MMTV/G-CSFR$^{-/-}$ mice. (D) Femurs of control, MT- or MT$^{G-CSF-/-}$-bearing mice. One-way ANOVA applied. Error bars represent SEM.
