# Supplementary material for: Tumor-derived GCSF Alters Tumor and Systemic Immune System Cell Subset Composition and Signaling
Source: Cancer Res Commun. 2023 Mar 9;3(3):404–19. doi: 10.1158/2767-9764.CRC-22-0278 (PMC9997410; doi:10.1158/2767-9764.CRC-22-0278)
Supplement: Figure S3 — Supplementary Figure S3 shows deep phenotyping of Neut/MDSCs and B cells in the bone marrow of mice bearing MT or MTG-CSF-/- tumors, using FlowSOM applied in tSNE maps. [file crc-22-0278-s05.pdf]

Figure S3

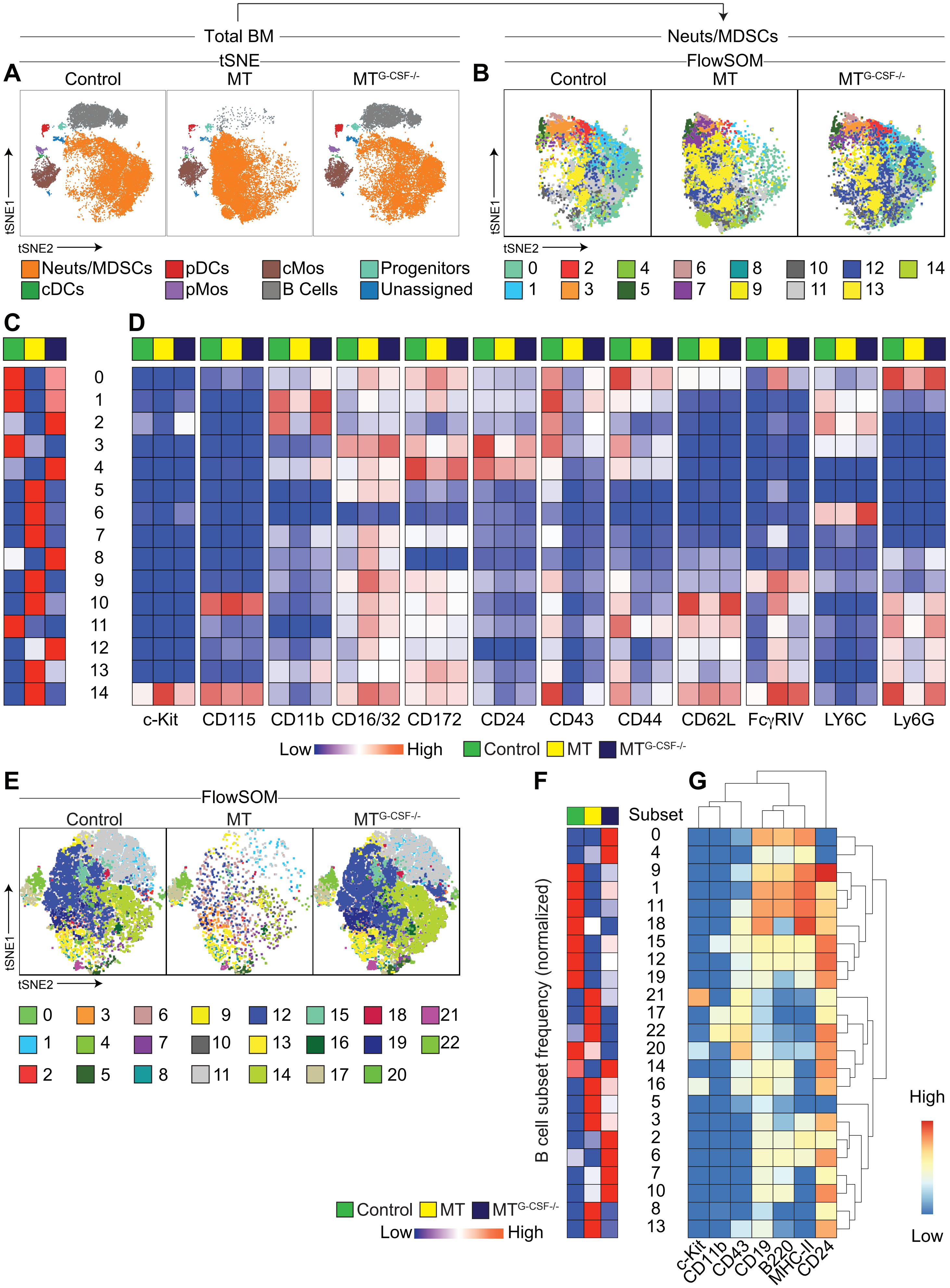

Total BM
Neuts/MDSCs
A
tSNE
Control
MT
MT$^{G-CSF-/-}$
tSNE1
tSNE2
Neuts/MDSCs
pDCs
cMos
Progenitors
cDCs
pMos
B Cells
Unassigned
B
FlowSOM
Control
MT
MT$^{G-CSF-/-}$
tSNE1
tSNE2
C
D
c-Kit
CD115
CD11b
CD16/32
CD172
CD24
CD43
CD44
CD62L
FcγRIV
LY6C
Ly6G
Low
High
Control
MT
MT$^{G-CSF-/-}$
E
FlowSOM
Control
MT
MT$^{G-CSF-/-}$
tSNE1
tSNE2
F
G
Subset
B cell subset frequency (normalized)
c-Kit
CD11b
CD43
CD19
B220
MHC-II
CD24
High
Low
Control
MT
MT$^{G-CSF-/-}$
Low
High

Figure S3. **Deep phenotyping of Neut/MDSCs and B cells in the bone marrow of tumor-bearing mice.** (A) tSNE maps of BM of healthy mice, or mice bearing MT or $MT^{G\text{-}CSF\text{-}/\text{-}}$ tumors. (B) FlowSOM was applied in tSNE maps to identify subpopulations of $CD11b^{+}$ $Ly6G^{+}$ Neut/MDSCs (orange population in A). (C) Changes in the proportion of each subpopulation of Neut/MDSC. (D) Means of indicated markers are shown for each Neut/MDSC subpopulation. (E) FlowSOM identified 23 subclusters of $Lineage^{-}$ $CD19^{+}$ B cells in BM samples of control, MT- or $MT^{G\text{-}CSF\text{-}/\text{-}}$-bearing mice (Grey population in A). (F) Frequency of subclusters shown in E. (G) Hierarchical clustering highlights differences in B cells subclusters in the BM of control, MT- or $MT^{G\text{-}CSF\text{-}/\text{-}}$-bearing mice. C, D, F Data were normalized (0-1 scale) per subpopulation.
