# Supplementary material for: Tumor-derived GCSF Alters Tumor and Systemic Immune System Cell Subset Composition and Signaling
Source: Cancer Res Commun. 2023 Mar 9;3(3):404–19. doi: 10.1158/2767-9764.CRC-22-0278 (PMC9997410; doi:10.1158/2767-9764.CRC-22-0278)
Supplement: Figure S4 — Supplementary Figure S4 shows that G-CSF and G-CSFR signaling inhibit DC development and activation, in DCs derived in a OP9-DLL1 co-culture system. [file crc-22-0278-s06.pdf]

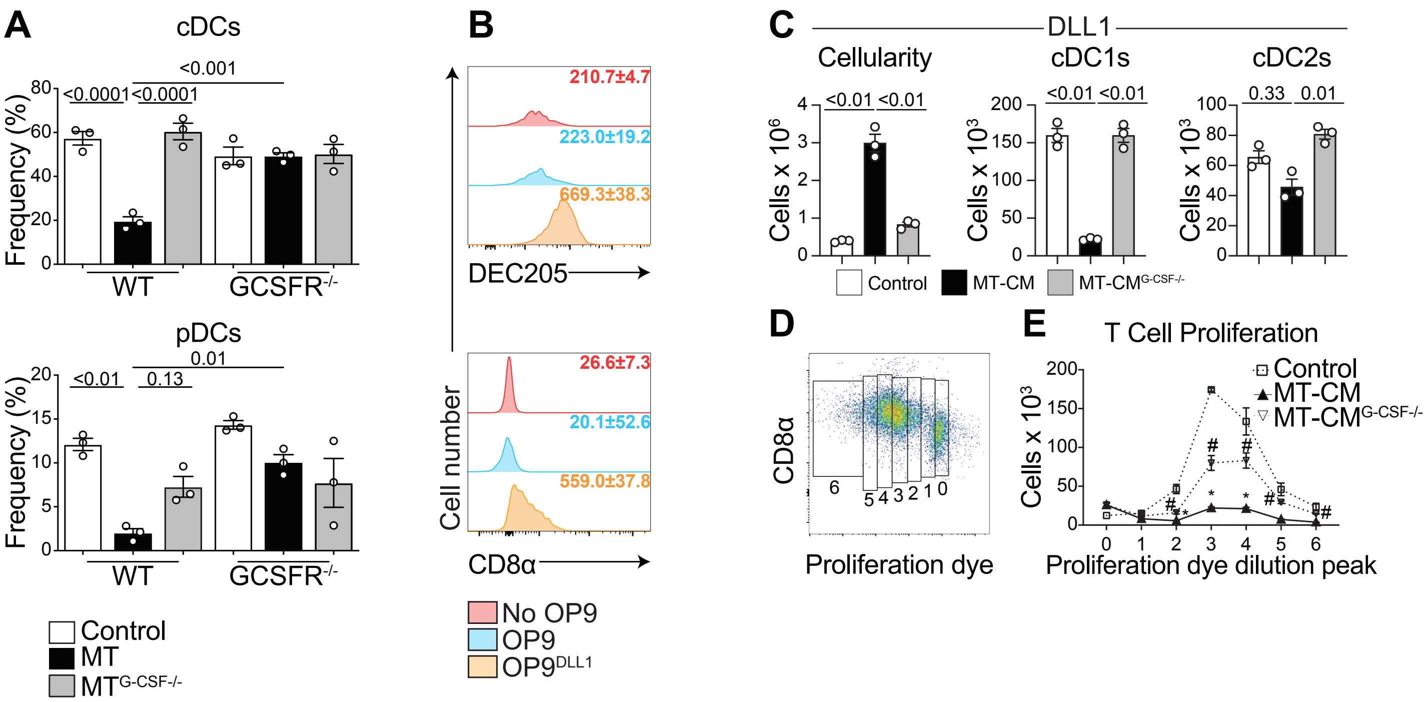

Figure S4. G-CSF and G-CSFR signaling impair DLL1-dependent DC development and activation *in vitro*. (A) Frequency of classic DCs and plasmacytoid DCs in Flt3L BM cultures (WT or G-CSFR$^{-/-}$) supplemented with 5% conditioned media from MT or MT$^{G\text{-}CSF-/-}$ tumor cells. (B) MFI of DEC205 and CD8α expression, two canonical markers expressed by *bone fide* mouse DCs, in BM Flt3L cultures grown in the presence or absence of OP9 (control) or OP9-DLL1 cell monolayers. (C) Quantification of total cells and cDC subsets in WT Flt3L BM cultures grown in the presence of OP9-DLL1 cell monolayer, +/- conditioned media from MT or MT$^{G\text{-}CSF-/-}$ tumor cells. (D) Dilution of proliferation dye in OT-I CD8^{+} T cells co-cultured with OVA-pulsed DC cultures for 72 hours. (E) Quantification of T cells in DC co-cultures. Dilution peaks are depicted in (D). Two-way ANOVA was used in E. One-way ANOVA applied elsewhere. Error bars represent SEM. * statistically significant comparing to control group; # statistically significant compared to MT-CM group.
